# Zhigancao decoction alleviates Parkinson’s disease via inhibiting TNF/NF-κB and Ras/ERK-mediated neuroinflammation and apoptosis

**DOI:** 10.1016/j.isci.2025.114489

**Published:** 2025-12-18

**Authors:** Jiakang Zhang, Xinlang Yu, Yuan Fang, Wenshan Li, Qi Cui, Xiaoyu Liu, Yanjie Jiang, Yin Zhang, Chengcheng Xu, Xin Sun, Yan Lu

**Affiliations:** 1 Department of Neurology, Nanjing Hospital of Chinese Medicine Affiliated to Nanjing University of Chinese Medicine, Nanjing, China; 2 Colorectal Surgery Center, Nanjing Hospital of Chinese Medicine Affiliated to Nanjing University of Chinese Medicine, Nanjing, China; 3 Department of Orthopedics, Nanjing Hospital of Chinese Medicine Affiliated to Nanjing University of Chinese Medicine, Nanjing, China; 4 Department of Gynecology, Nanjing Hospital of Chinese Medicine Affiliated to Nanjing University of Chinese Medicine, Nanjing, China

**Keywords:** Biological sciences, Ethnopharmacology, Phytopharmacy

## Abstract

Parkinson’s disease (PD) is characterized by the progressive loss of dopaminergic neurons, with neuroinflammation and apoptosis serving as key pathological drivers. In this study, we investigated the traditional herbal formula Zhigancao Decoction (ZGCD) using an integrated strategy that combined network pharmacology prediction, experimental validation in MPTP-induced mouse and MPP^+^-treated cellular models, and molecular docking simulation. Our results demonstrate that ZGCD alleviates motor deficits and protects dopaminergic neurons by concurrently inhibiting the TNF/NF-κB and Ras/ERK signaling pathways, thereby reducing the release of pro-inflammatory cytokines and markers of apoptosis. *In vitro* experiments further confirmed that the bioactive components apigenin and bisdemethoxycurcumin contribute to this multi-pathway regulatory effect. This systems pharmacology approach elucidates how ZGCD simultaneously targets interconnected disease-relevant pathways, highlighting its potential as a multi-target therapeutic strategy for neurodegenerative disorders.

## Highlights

• ZGCD ameliorates motor dysfunction in MPTP-induced Parkinson’s disease mice
• ZGCD protects dopaminergic neurons by mitigating neuroinflammation and apoptosis
• The mechanism involves the dual inhibition of the TNF/NF-κB and Ras/ERK pathways
• Active compounds apigenin and bisdemethoxycurcumin contribute to these effects

## Graphical abstract

**Figure:**
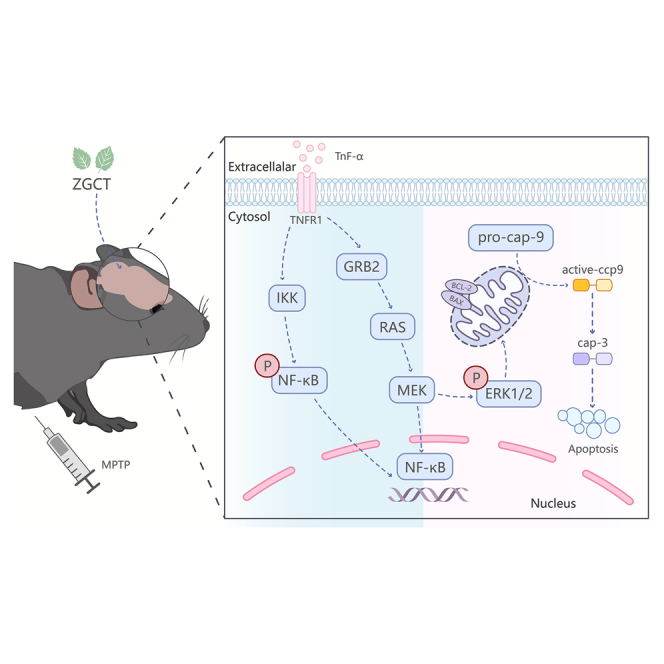

## Introduction

Parkinson’s disease (PD) represents the second most prevalent neurodegenerative disorder worldwide. Its pathological hallmarks primarily involve the progressive degeneration of dopaminergic (DA) neurons in the substantia nigra pars compacta (SNpc) and striatal DA denervation, leading to severe motor dysfunction.^1^ The clinical presentation of PD is primarily characterized by motor impairments, including bradykinesia, resting tremor, gait abnormalities, and postural instability. The underlying pathogenesis involves α-synuclein (α-syn) aggregation, neuroinflammatory responses, apoptotic pathways, oxidative stress cascades, and mitochondrial dysfunction.^2^ Current therapeutic strategies for PD are categorized into surgical interventions and pharmacological treatments. Pharmacotherapy remains the cornerstone of PD treatment. However, substantial interindividual variability in drug bioavailability and suboptimal therapeutic outcomes remain as major clinical challenges. Moreover, prolonged medication administration frequently induces adverse effects that significantly compromise patients’ quality of life.^3^ Therefore, effective therapeutic strategies that durably enhance PD patients’ quality of life remain critically needed.^4^

Contemporary perspectives increasingly recognize PD as a multisystem disorder characterized by prominent neuroinflammation and immune dysregulation. While neuroinflammatory processes may not constitute the primary etiological factor in all PD cases, accumulating evidence positions neuroinflammation as a critical disease-modifying mechanism facilitating pathological progression.^5^ The interplay between programmed cell death and neurodegenerative pathology remains intimately connected, with apoptosis constituting the predominant neuronal demise mechanism in PD. Chronic inflammatory milieus perpetuate cytokine-mediated apoptotic cascades, ultimately resulting in irreversible neuronal degeneration and functional impairment of cerebral networks.^6^

Traditional Chinese Medicine (TCM), constituting a significant component of global pharmacotherapy, exhibits a multi-component nature, multi-target mechanisms, and multi-pathway therapeutic effects with favorable safety profiles, thereby offering promising alternatives for PD management.^7^^,^^8^ TCM contains abundant natural chemical constituents. It demonstrates therapeutic potential through the modulation of cellular signaling pathways, attenuation of neuroinflammation and oxidative stress, and regulation of programmed cell death processes.^9^ Zhigancao Decoction (ZGCD) is a canonical herbal formulation. It was originally documented in the *Shang Han Lun*, a classic foundational text of Chinese medicine. Historically, it has been used in cardiovascular therapeutics. ZGCD is a polyherbal formulation. It contains nine medicinal components: *rehmannia, Licorice, Ginseng, Jujube, donkey-hide gelatin, Ophiopogon, cassia twig, Ginger, Fructus Cannabis.* This herbal complex exerts multiple neuroprotective effects. These benefits arise from its diverse phytochemical constituents. ZGCD includes pharmacologically active flavonoids, phenolic acids, alkaloids, and terpenoids, which collectively exhibit anti-inflammatory, antioxidant, and neuroprotective properties. Experimental evidence suggests that ZGCD-mediated therapeutic effects may involve the attenuation of neuroinflammatory responses, mitigation of neuronal oxidative damage, and counteraction of synaptic dysfunction. Particularly noteworthy is its capacity to ameliorate DA neuronal degeneration, thereby potentially alleviating neurodegenerative manifestations associated with PD.^10^ The neuroprotective efficacy of ZGCD constituents can be mechanistically delineated through their distinct bioactive components. Glycyrrhizin, a predominant triterpenoid in Glycyrrhizae Radix Praeparata, demonstrates therapeutic potential in 1-methyl-4-phenyl-1,2,3,6-tetrahydropyridin (MPTP)-induced murine models by attenuating neuroinflammatory cascades, oxidative stress burden, and autophagic flux dysregulation, thereby preserving neuronal integrity. Ginsenoside Rg1, a protopanaxatriol-type saponin isolated from Ginseng Radix, has been shown to ameliorate autophagic degradation impairments of RTP801 and α-syn aggregates, significantly improving motor dysfunction in PD animal models.^11^^,^^12^ Catalpol, an iridoid glycoside derived from Rehmanniae Radix, exhibits blood-brain barrier-permeable properties and serves as a neuroprotective agent in neurodegenerative disorders, particularly through preserving DA neuronal viability via the modulation of mitochondrial homeostasis.^13^ Cinnamomi Ramulus contributes cinnamic acid derivatives that effectively inhibit the amyloidogenic transformation of α-syn, suggesting therapeutic applicability in synucleinopathies including PD.^14^ Experimental investigations on Zingiberis Rhizoma Recens reveal that its active constituents (zingerone, 6-shogaol, and 6-gingerol) exert multimodal anti-PD effects through the coordinated regulation of neuroinflammatory responses, redox equilibrium, and intestinal barrier integrity.^15^^,^^16^ Therapeutic synergism arising from the nine-herb combinatorial architecture of ZGCD likely operates through multi-target mechanisms encompassing anti-inflammatory signaling, oxidative damage mitigation, and apoptosis inhibition. This phytopharmaceutical synergy underscores ZGCD’s potential as a disease-modifying intervention in PD pathogenesis.

However, the therapeutic effects of ZGCD on PD pathogenesis and progression remain mechanistically unexplored. Based on the above theories and pharmacological foundations, this study was designed to elucidate the therapeutic effects and mechanisms of ZGCD in PD models. This study elucidated the molecular mechanisms underlying ZGCD therapeutic effects against PD through an integrated methodology combining network pharmacology, UPLC-MS/MS analysis, *in vivo* and *in vitro* validation, and molecular docking simulations. These findings demonstrate TCM potential as an adjunctive therapeutic strategy for PD management.

## Results

### Network pharmacology predicted the potential mechanism of Zhigancao Decoction on Parkinson’s disease

**Figure 1:**
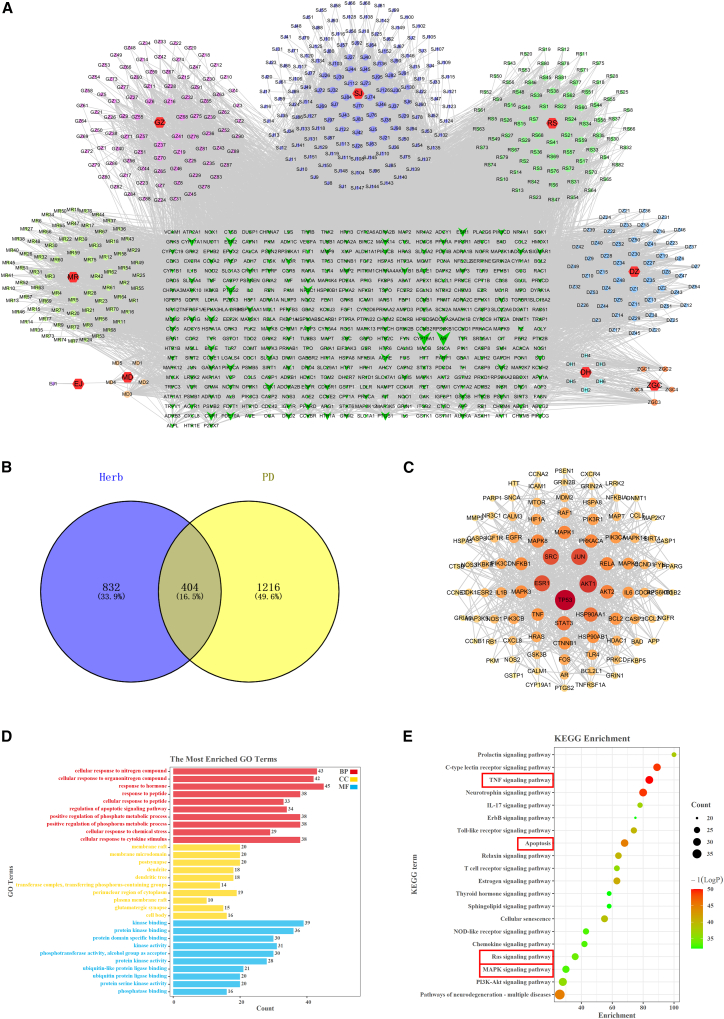
Network pharmacology predicted that ZGCD treatment for Parkinson’s disease might be associated with apoptosis, inflammation, TNF/NF-κB, and Ras/ERK signaling pathways (A) ZGCD Herbal Active Ingredient-Target Network. (B) Venn diagrams of active ingredients and disease targets. (C) The PPI network of the common targets of ZGCD and PD. (D) GO enrichment analysis of potential therapeutic targets. (E) Enrichment analysis of KEGG pathways for potential therapeutic targets.

To elucidate the molecular mechanisms underlying ZGCD therapeutic effects in PD, 736 bioactive phytocompounds were systematically retrieved through the TCMSP and HERB databases. Subsequently, 1,236 potential compound targets were identified, and a comprehensive herbal-compound-target interaction network was constructed (Figure 1A). Meanwhile, 1,620 PD-related targets were collected using multiple disease databases. We compared the targets related to ZGCD and PD, obtained 404 potential targets shared by ZGCD and PD, and obtained 95 important targets through median screening (Table S4) and plotted the Venn diagram (Figure 1B). Import 95 key targets into the STRING database to draw the PPI network (Figure 1C), The core targets, such as TP53, SRC, ESR1, JUN, AKT1, MAPK3, MAPK1, TNF, and NF-κB, were analyzed and determined. It was found that they were closely related to the targets such as IL-6, Bcl-2, IL-1β, and Caspase-3. These targets may be crucial in the anti-PD effect exerted by ZGCD. Through GO enrichment analysis, the possible biological mechanisms by which ZGCD regulates PD were further discovered. These targets are involved in processes such as apoptosis, inflammatory responses, and the regulation of protein phosphorylation (Figure 1D). KEGG pathway enrichment analysis further highlighted the significant involvement of these targets in neurodegeneration-associated signaling cascades, notably including the MAPK signaling pathway, Ras signaling transduction, TNF pathway, apoptotic regulation, and inflammatory response, among other critical pathological networks (Figure 1E; Table S5).

### Quality control of Zhigancao Decoction

**Figure 2:**
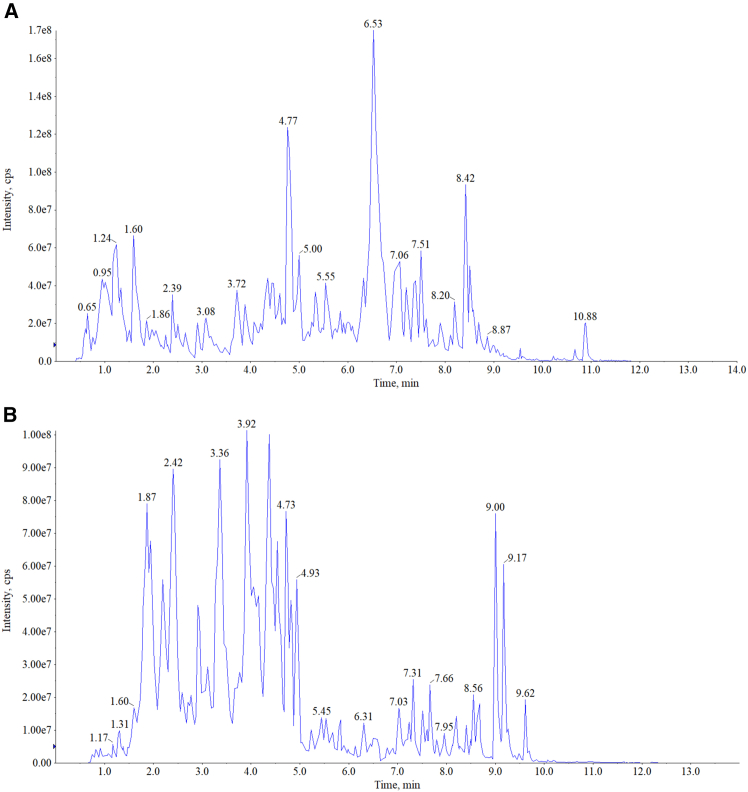
The quality control of ZGCD was carried out through UPLC-MS/MS (A and B) Total ion chromatograms (TICs) of Z/∗GCD in positive (A) and negative (B) ion modes.

We determined the main chemical substances in the ZGCD sample through UPLC-MS/MS analysis. Figures 2A and 2B, respectively, show the TIC diagrams in the positive ion and negative ion flow modes. A total of 1,720 distinct compounds were identified, comprising 405 flavonoids, 315 phenolic acids, 277 terpenoids, 248 alkaloids, 105 lignans and coumarins, 39 steroids, 28 quinones, 7 tannins, and 296 compounds from other chemical classes. The main compounds included are shown in Table 2, such as 3-Hydroxybenzoate, Protocatechuic acid-4-*O*-glucoside, 1-*O*-Gentisoyl-β-D-glucoside. The supplementary table provides comprehensive details of the 1,720 compounds identified in ZGCD, including their chemical classifications, structural identifiers, and phytochemical properties (Table S3).

**Table 2:** Main chemical components of ZGCD

Compounds	Class	Formula	Molecular weight (Da)	Ionization model	Relative content
3-Hydroxybenzoate	Phenolic acids	C7H6O3	138.0317	[M-H]-	179946167.6
Protocatechuic acid-4-O-glucoside	Phenolic acids	C13H16O9	316.0794	[M-H]-	123537810.2
1-O-Gentisoyl-β-D-glucoside	Phenolic acids	C13H16O9	316.0794	[M-H]-	123537810.2
Passibiflorin	Alkaloids	C18H27NO11	433.1584	[M+H]+	120323729.7
Naringenin-4'-O-glucoside	Flavonoids	C21H22O10	434.1213	[M-H]-	118329891.3
Heptane-1,7-diol sulfate	Others	C7H16O5S	212.0713	[M-H]-	104071316.1
6-O-Feruloylajugol	Terpenoids	C25H32O12	524.1894	[M-H]-	97650428.9
Sophoflavescenol	Flavonoids	C21H20O6	368.126	[M+H]+	85389381.7
macedonic acid-GlcA	Terpenoids	C36H54O10	646.3717	[M+H]+	78323376.1
naringenin 5-glucoside	Flavonoids	C21H22O10	434.1213	[M-H]-	68716540.0
L-Histidinol	Alkaloids	C6H11N3O	141.0902	[M+H]+	67911295.7
Verbascoside	Phenolic acids	C29H36O15	624.2054	[M-H]-	66237074.2
2-Amino-4-methylvaleric acid	Others	C6H13NO2	131.0946	[M+H]+	64201002.0
(2S)-2-amino-2-methylpentanoic acid	Alkaloids	C6H13NO2	131.0946	[M+H]+	63422862.6
Coumaroyl Quinic Acid	Others	C16H18O8	338.1002	[M-H]-	63063217.6
3-O-p-Coumaroylquinic acid	Phenolic acids	C16H18O8	338.1002	[M-H]-	63063217.6
1-O-p-Coumaroylquinic acid	Phenolic acids	C16H18O8	338.1002	[M-H]-	61435004.4
2,3-Dihydroxybenzoate	Phenolic acids	C7H6O4	154.0266	[M-H]-	60484073.3
2,5-Dihydroxybenzoate	Phenolic acids	C7H6O4	154.0266	[M-H]-	60484073.3
[6]-Shogaol	Phenolic acids	C17H24O3	276.1725	[M+H]+	56567375.1
Isomartynoside	Phenolic acids	C31H40O15	652.2367	[M-H]-	53470938.0
N-Caffeoyltyramine	Alkaloids	C17H17NO4	299.1158	[M+H]+	52804852.9
3,4-Dihydroxybenzoate	Phenolic acids	C7H6O4	154.0266	[M-H]-	51740500.3
Martynoside	Phenolic acids	C31H40O15	652.2367	[M-H]-	50347759.5
Geniposidic acid	Terpenoids	C16H22O10	374.1213	[M-H]-	49628167.9
3,3',4',5,6,7,8-heptamethoxyflavone	Flavonoids	C22H24O9	432.142	[M+H]+	49193626.3
4-(3-O-Sulfo-Beta-D-Glucopyranosyloxy) -3-Hydroxybenzoic Acid	Phenolic acids	C13H16O12S	396.0362	[M-H]-	49075435.3
gnetifolin E	Others	C21H24O9	420.142	[M-H]-	48366524.9
Rehmannioside D	Terpenoids	C27H42O20	686.2269	[M-H]-	47525545.9
Caffeate	Phenolic acids	C9H8O4	180.0423	[M-H]-	46801111.4

### Zhigancao Decoction improves behavioral and motor deficits in 1-methyl-4-phenyl-1,2,3,6-tetrahydropyridin mice

**Figure 3:**
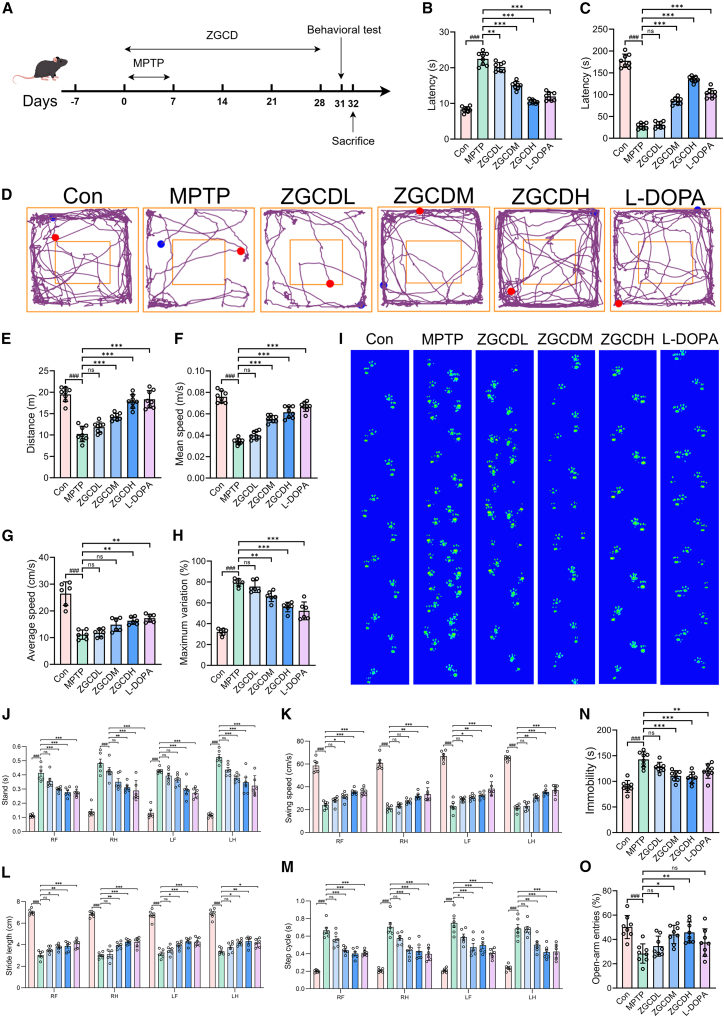
ZGCD treatment can alleviate behavioral and motor deficits in MPTP-Induced PD mice (A) Schematic diagram of the MPTP-induced PD mouse model (This figure was drawn by Figdraw). (B) The pole test recorded the time required for the mice to come down from the pole (*n* = 8). (C) The time required for mice to walk on a cylindrical rotating rod was recorded in the rotarod test (*n* = 8). (D) Representative trajectory images of the mice indicated by the open field test. (E) The total 5 min walking distance of the mice was specified in the open field test (*n* = 8). (F) The average speed of the mice referred to in the open field test (*n* = 8). (G) Evaluate the average speed through gait analysis (*n* = 6). (H) The maximum variation was evaluated through gait analysis (*n* = 6). (I) Visualize the gait pattern as a footprint view. RF, right front. RH, right hind. LF, left front. LH, left hind. (J–M) assesses motor coordination disorders in mice through gait analysis, including standing (J), swing speed (K), stride length (L), and step cycle (M) (*n* = 6). (N) Immobility time in FST (*n* = 8). (O) open arms entries in EPM (*n* = 8). ###*p* < 0.001 vs. Con; ∗*p* < 0.05, ∗∗*p* < 0.01 and ∗∗∗*p* < 0.001 vs. MPTP. Data are expressed as mean ± SD.

We conducted *in vivo* studies using the MPTP mouse model (Figure 3A). Behavioral tests, including the open field, pole, and rotarod tests, were employed to compare motor function between control mice and MPTP-induced PD mice. The PD model group exhibited significant motor deficits (Figures S1C–S1F). Concurrently, immunohistochemical analysis revealed a marked reduction in the number of TH^+^ neurons in the SNpc of MPTP-treated mice compared to controls, which is a hallmark pathological feature of PD (Figures S1A and S1B). Collectively, these behavioral and histological findings confirm the successful establishment of the PD model. Four behavioral tests were carried out to evaluate the efficacy of ZGCD in motor symptoms, including the pole test, the rotarod test, the open field test, and gait analysis. The behavioral detection results are shown in Figure 3, confirming that the motor function of the MPTP model mice has been significantly impaired. The pole test and the rotarod test can effectively detect the motor coordination ability of mice. In the pole test, MPTP-treated mice exhibited significantly prolonged latency to completely turn and descend from the top to the base of the pole compared to the control group. ZGCD administration demonstrated dose-dependent improvements, substantially reducing descent latency, with the high-dose group showing the most pronounced efficacy (Figure 3B). Rotarod performance analysis revealed that MPTP-induced mice displayed markedly impaired motor coordination, evidenced by shortened fall latency. While the ZGCDL group showed no significant improvement, high-dose ZGCD treatment significantly extended the duration of balance maintenance on the rotating rod (Figure 3C). The open field test mainly reflects the autonomous activity ability of mice. Analysis of open field trajectory plots revealed that MPTP-induced PD mice exhibited significant reductions in exploratory distance traveled in both peripheral and central zones compared to the control group. While the low-dose ZGCD cohort showed no substantial improvement, medium and high-dose ZGCD administration significantly increased exploratory distance in both field compartments (Figure 3D). Quantitatively, MPTP mice demonstrated markedly decreased total locomotion distance and reduced average velocity versus controls. The low-dose ZGCD group failed to ameliorate these deficits, whereas medium-dose treatment partially restored motor parameters. Notably, high-dose ZGCD and L-DOPA groups exhibited significantly greater total distance traversed and higher average velocity (Figures 3E and 3F). It indicates that the motor ability has been significantly improved. Gait parameters were quantitatively assessed using the CatWalk system, which records paw-print impressions during spontaneous ambulation across a glass walkway. Key metrics analyzed included stance phase duration, swing speed, stride length, gait cycle interval, mean velocity, and maximum coefficient of variation of stride time. Footprint maps provided visual evidence of locomotor integrity: MPTP-treated mice exhibited significantly disorganized paw placement patterns compared to controls, whereas medium/high-dose ZGCD and L-DOPA groups demonstrated restored footprint coherence. Quantitative gait analysis demonstrated that MPTP-treated mice exhibited significantly reduced mean velocity traversing the walkway, indicating substantial motor impairment. Concurrently, the maximum stride time coefficient of variation increased, confirming disrupted step regularity and impaired motor coordination (Figures 3G–3M). These results demonstrate that ZGCD ameliorates gait disturbances in MPTP-induced PD mice, with therapeutic efficacy exhibiting dose-dependent characteristics. Collectively, this evidence robustly demonstrates that ZGCD treatment ameliorates PD motor symptoms. Furthermore, we evaluated anxiety- and depressive-like behaviors in PD mice using the FST and EPM. The results demonstrated that MPTP-treated PD mice exhibited significantly increased anxiety and depressive-like behaviors, while ZGCD administration significantly alleviated these behavioral abnormalities in a dose-dependent manner (Figures 3N and 3O).

### The effects of Zhigancao Decoction on TH and α-synuclein expression in 1-methyl-4-phenyl-1,2,3,6-tetrahydropyridin-induced Parkinson’s disease mice

**Figure 4:**
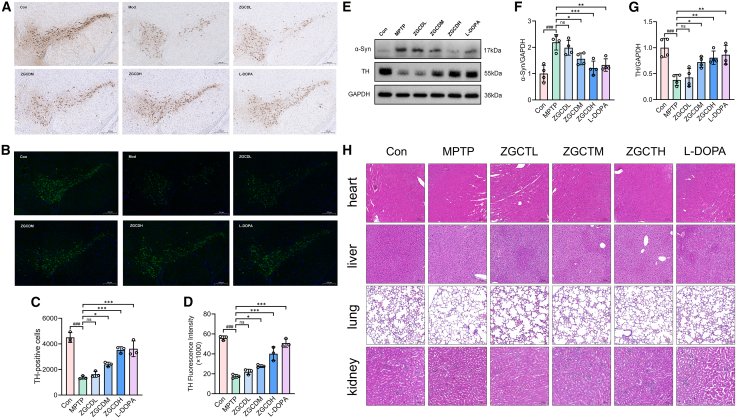
ZGCD treatment can alleviate the degeneration of DA neurons (A) IHC analysis of TH expression in SNpc. Scale bars, 200 μm. (B) Immunostaining analysis of TH expression in SNpc (green). DAPI (blue) is used for identifying cell nuclei. Scale bars, 200 μm. (C) Quantitative analysis of TH-positive neurons in SNpc (*n* = 3). (D) Quantification of relative TH strength in SNpc (*n* = 3). (E) WB was used to detect the representative expressions of TH and α-Syn in SN. (F and G) Statistics of the relative expression level of α-Syn and TH in SN (*n* = 4). (H) HE staining of heart, liver, lung, and kidney tissues to examine pathological changes in mice. Scale bars, 100 μm ###*p* < 0.001 vs. Con; n.s., not significant, ∗*p* < 0.05, ∗∗*p* < 0.01 and ∗∗∗*p* < 0.001 vs. MPTP. Data are expressed as mean ± SD.

Degeneration of DA neurons, resulting in diminished dopamine synthesis, constitutes a central pathological mechanism in PD pathogenesis.^17^ As the rate-limiting enzyme in dopamine biosynthesis, TH governs DA neurotransmission efficacy. TH critically determines striatal dopamine production through its expression dynamics.^18^ Pathogenic α-syn aggregates negatively regulate TH enzymatic activity and modulate dopamine transporter function, thereby disrupting both dopamine biosynthesis and synaptic reuptake processes.^19^ To evaluate the neuroprotective effects of ZGCD, we quantified pathological biomarkers in the SN using IHC and IF analyses. MPTP-induced mice exhibited significantly reduced TH expression in the SN, whereas ZGCD treatment substantially restored TH immunoreactivity to near-physiological levels (Figures 4A–4D). Additionally, WB analysis was performed to quantify TH and α-syn protein expression in the SN of experimental mice. The results confirmed significant downregulation of TH expression and concurrent upregulation of α-syn accumulation in MPTP-treated mice compared to controls. High-dose ZGCD effectively prevented TH depletion and suppressed pathological α-syn aggregation. In contrast, ZGCDL and ZGCDM group regimens demonstrated suboptimal efficacy in modulating these protein alterations (Figures 4E–4G). Furthermore, histopathological examination via HE staining revealed no significant structural alterations in cardiac, hepatic, pulmonary, or renal tissues following 4 weeks of ZGCD treatment, demonstrating the absence of overt toxic effects on major organs within the established dosing regimen (Figure 4H). Collectively, these results demonstrate that ZGCD attenuates DA neuronal degeneration in PD murine models, reflecting its neuroprotective efficacy against this core pathological hallmark of PD.

### Zhigancao Decoction mitigates inflammation to protect dopaminergic neurons from 1-methyl-4-phenyl-1,2,3,6-tetrahydropyridin-induced apoptosis *in vivo*

**Figure 5:**
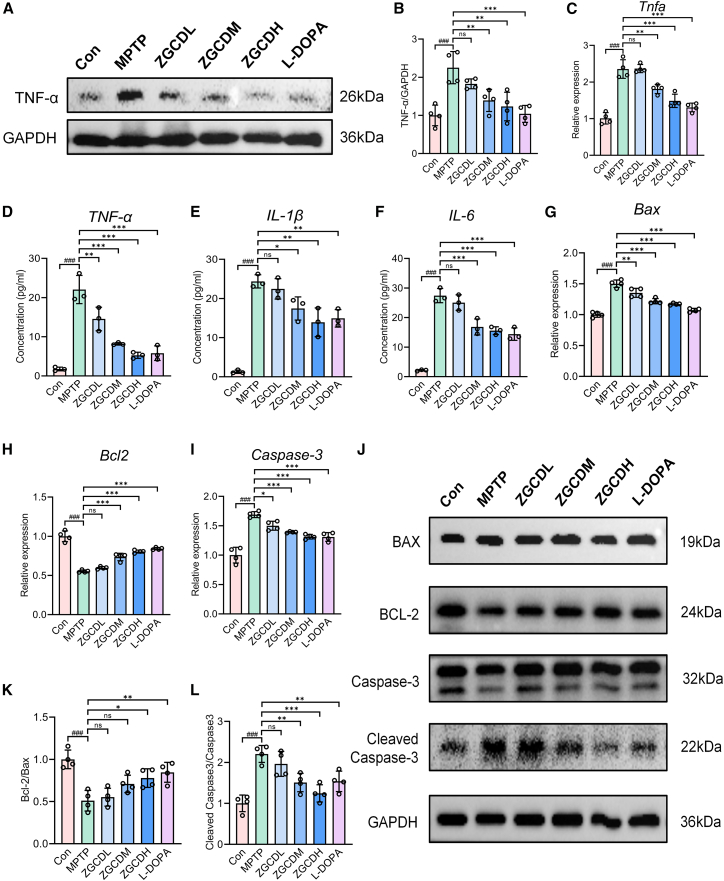
ZGCD alleviates inflammation and apoptosis in PD mice induced by MPTP (A) WB was used to detect the representative expressions of TNF-α in SN. (B) Statistics of the relative expression level of TNF-α in SN (*n* = 4). (C) Representative expression of *TNF-α* detected by RT-qPCR. (D–F) The levels of TNF-α, IL-6, and IL-1β were detected by ELISA (*n* = 3). (G–I) Representative expression of *Bax*, *Bcl-2 and Caspase-3* detected by RT-qPCR (*n* = 4). (J) WB was used to detect the representative expressions of Bax, Bcl-2, Cleaved Caspase-3, and Caspase-3 in SN. (K and L) Statistics of the relative expression level of Bcl-2/Bax and Cleaved Caspase-3/Caspase-3 in SN (*n* = 4). ###*p* < 0.001 vs. Con; n.s., not significant, ∗*p* < 0.05, ∗∗*p* < 0.01 and ∗∗∗*p* < 0.001 vs. MPTP. Data are expressed as mean ± SD.

To investigate the neuroprotective effects of ZGCD on DA neurons in PD murine models, we analyzed inflammatory and apoptotic biomarkers in the SN. WB and RT-qPCR analysis demonstrated significantly elevated TNF-α levels in MPTP-treated mice compared to controls, whereas ZGCD administration substantially suppressed TNF-α expression (Figures 5A–5C). Furthermore, ELISA assays revealed enhanced neuroinflammatory responses in MPTP mice, characterized by the marked upregulation of pro-inflammatory cytokines (TNF-α, IL-1β, and IL-6) in nigral tissue. Critically, ZGCD treatment downregulated the expression of these pathogenic mediators in a dose-dependent manner (Figures 5D–5F). Concurrently, MPTP administration markedly enhanced the expression of apoptosis-associated proteins and genes in the SN. WB analysis quantified key apoptotic regulators—including pro-apoptotic Bax, anti-apoptotic Bcl-2, Caspase-3, and its activated form Cleaved Caspase-3. MPTP-treated mice exhibited significantly reduced Bcl-2/Bax ratio, and increased Cleaved Caspase-3/Caspase-3 ratio. High-dose ZGCD profoundly attenuated these apoptotic alterations, normalizing Bcl-2 expression and suppressing Caspase-3 activation (Figures 5J–5L). Complementary RT-qPCR analysis further confirmed MPTP-induced transcriptional upregulation of Bax and Caspase-3, while ZGCD treatment significantly downregulated these pro-apoptotic genes in a dose-dependent manner (Figures 5G–5I). Collectively, these findings indicate that ZGCD may exert neuroprotective effects by attenuating neuroinflammatory cascades and mitigating apoptotic pathways in DA neurons.

### Zhigancao Decoction alleviates 1-methyl-4-phenyl-1,2,3,6-tetrahydropyridin-induced dopaminergic neuronal damage by regulating the TNF/NF-κB and Ras/ERK pathway *in vivo*

**Figure 6:**
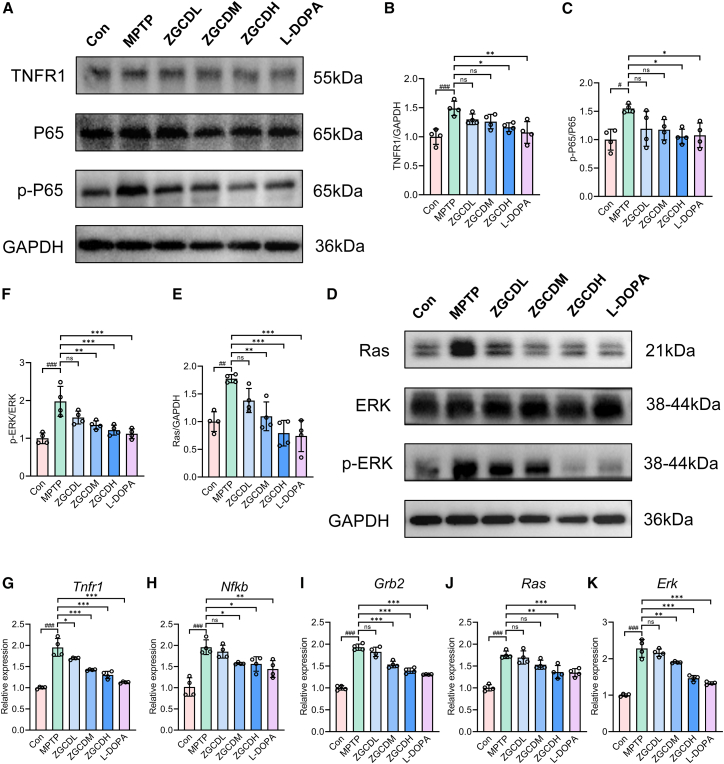
ZGCD treatment may alleviate MPTP-induced injury of SN DA neurons by inhibiting the TNF/NF-kB and Ras/ERK pathways (A) WB was used to detect the representative expressions of TNFR1, P65, and p-P65 in SN. (B and C) Statistics of the relative expression level of TNFR1 and p-P65/P65 in SN (*n* = 4). (D) WB was used to detect the representative expressions of Ras, ERK, and *p*-ERK in SN. (E and F) Statistics of the relative expression level of Ras and *p*-ERK/ERK in SN (*n* = 4). (G–K) Representative expression of *Tnfr1, Nfkb, Grb2, Ras,* and *Erk* detected by RT-qPCR (*n* = 4). ###*p* < 0.001 vs. Con; n.s., not significant, ∗*p* < 0.05, ∗∗*p* < 0.01 and ∗∗∗*p* < 0.001 vs. MPTP. Data are expressed as mean ± SD.

Dysregulation of the TNF/NF-κB and Ras/ERK signaling pathways plays a pivotal role in PD pathogenesis and is strongly implicated in other neurodegenerative disorders.^20^ KEGG enrichment analysis suggested that ZGCD likely exerts anti-PD effects through the modulation of these pathways. To investigate whether the ameliorative effects of ZGCD on MPTP-induced neuroinflammation and apoptosis are mediated by TNF/NF-κB and Ras/ERK signaling, we performed WB and qPCR to quantify pathway-related protein and gene expression in the murine SN. WB analysis revealed elevated levels of TNFR1 and p-P65 in the SN of MPTP-treated mice. Compared to the MPTP group, ZGCD administration significantly suppressed the expression of key TNF/NF-κB signaling proteins (Figures 6A–6C). Furthermore, increased expression of Ras and *p*-ERK was observed in the nigral tissue of MPTP mice, whereas ZGCD treatment dose-dependently inhibited the activation of these pathway effectors (Figures 6D–6F). Concomitantly, RT-qPCR analysis substantiated the transcriptional upregulation of *Tnfr1*, *Nfkb*, *Grb2*, *Ras*, and *Erk* genes in the SN of MPTP-treated mice. ZGCD intervention attenuated this aberrant gene expression profile, effectively normalizing pathway-associated transcript levels toward physiological baselines (Figures 6G–6K). Collective experimental evidence conclusively demonstrates that ZGCD confers neuroprotection by suppressing the MPTP-induced hyperactivation of the TNF/NF-κB and Ras/ERK signaling pathways, thereby attenuating downstream neuroinflammatory and apoptotic cascades in DA neurons.

### ZGCDS alleviates 1-methyl-4-phenylpyridinium^+^-induced inflammation and attenuates 1-methyl-4-phenylpyridinium^+^-induced apoptosis in SH-SY5Y cells

**Figure 7:**
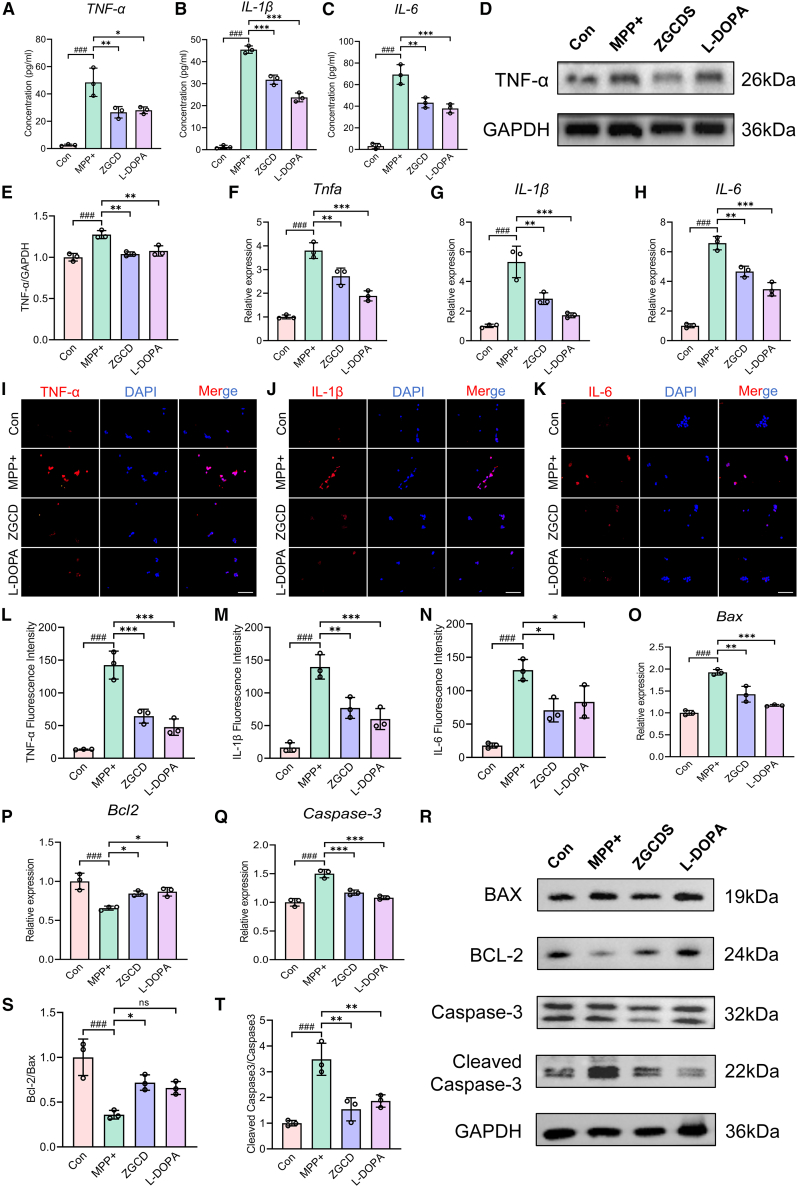
ZGCDS treatment may alleviate MPTP-induced injury of SN DA neurons by inhibiting the TNF/NF-kB and Ras/ERK pathways (A–C) The levels of TNF-α, IL-1β, and IL-6 were detected by ELISA in SH-SY5Y cells (*n* = 3). (D) WB was used to detect the representative expressions of TNF-α in SH-SY5Y cells. (E) Statistics of the relative expression level of TNF-α in SH-SY5Y cells (*n* = 3). (F–H) Representative expression of *TNF-α, IL-1β,* and *IL-6* detected by RT-qPCR (*n* = 3). (I–N) IF was used to analyze the expressions of TNF-α, IL-1β, and IL-6 in SH-SY5Y cells (*n* = 3). Scale bars, 100 μm. (O–Q) Representative expression of *Bax, Bcl-2,* and *caspase-3* detected by RT-qPCR in SH-SY5Y cells (*n* = 3). (R–T) WB analysis of Bax, Bcl-2, Caspase-3, and Cleaved Caspase-3 expression in SH-SY5Y cells (*n* = 3). ###*p* < 0.001 vs. Con; n.s., not significant, ∗*p* < 0.05, ∗∗*p* < 0.01 and ∗∗∗*p* < 0.001 vs. MPTP. Data are expressed as mean ± SD.

We evaluated the cytotoxic effect of MPP^+^ on the viability of SH-SY5Y cells through the CCK-8 assay and observed a significant reduction in cell viability at a concentration of 1 mM (Figure S1G). We next treated the cells with varying concentrations of MPP^+^ and measured the expression of the inflammatory cytokine TNF-α by ELISA and the pro-apoptotic protein Bax by WB. The results showed that at 1 mM MPP^+^, the expression of both TNF-α and Bax was significantly increased, indicating the marked induction of inflammatory and apoptotic responses (Figures S1H–S1J). Furthermore, no statistically significant differences in the expression levels of TNF-α or Bax were observed between the 1.0 mM and 2.0 mM MPP^+^ treatment groups. Therefore, in the subsequent cell experiments, we chose the 1 mM MPP^+^ cell model for the experiments. Furthermore, the CCK-8 assay revealed that treatment with varying concentrations of ZGCDS (ranging from 5% to 25%) showed no significant cytotoxicity toward SH-SY5Y cells (Figure S1K). Subsequently, MPP^+^-injured cells were treated with different concentrations of ZGCDS within this range. The results demonstrated that 20% ZGCDS produced the most pronounced therapeutic effect (Figure S1L). Therefore, in the subsequent experiments, we selected 20% concentration of drug-containing serum to explore the protective effect of ZGCDS on MPP^+^-induced cell damage. Neuroinflammation and apoptotic cascades play pivotal roles in the pathogenesis of PD, significantly contributing to PD-associated neurodegeneration.^21^ ELISA quantification revealed elevated intracellular levels of pro-inflammatory cytokines (TNF-α, IL-6, and IL-1β) in MPP^+^-exposed SH-SY5Y cells (Figures 7A–7C). WB analysis confirmed significant upregulation of TNF-α protein expression in the neurotoxic model group, while RT-qPCR and IF assays validated transcriptional and translational amplification of pro-inflammatory mediators (Figures 7D–7N). Crucially, treatment with ZGCDS significantly downregulated these inflammatory cytokine levels. WB analysis revealed that MPP^+^-exposed cells exhibited a significant decrease in the anti-apoptotic Bcl-2/Bax ratio and elevated Cleaved Caspase-3 expression. Crucially, ZGCDS treatment reversed these apoptotic alterations, increased Bcl-2/Bax and suppressed Caspase-3 activation (Figures 7R–7T). Concomitant RT-qPCR validation confirmed concordant transcriptional regulation of *Bcl-2*, *Bax*, and *Caspase-3* genes (Figures 7O–7Q). Synthesizing the aforementioned experimental evidence, we conclude that ZGCDS confers neuroprotection to DA neurons by significantly attenuating MPP^+^-induced neuroinflammatory responses and mitochondrial apoptotic pathways.

### ZGCDS protects SH-SY5Y cells by regulating the TNF/NF-kB and Ras/ERK signaling pathway

**Figure 8:**
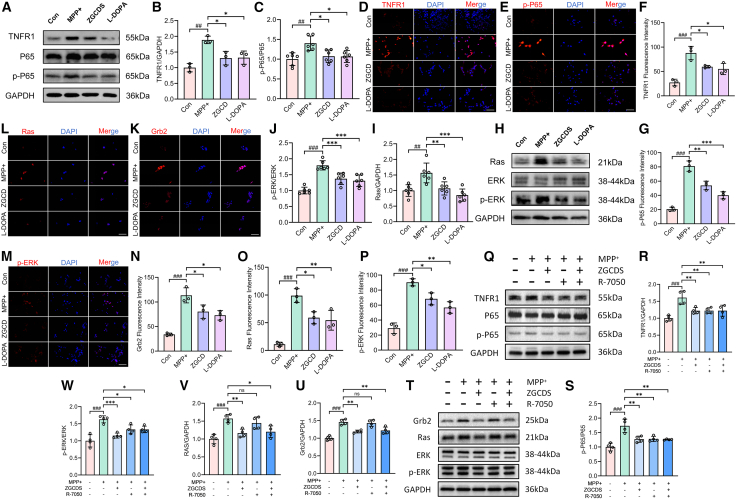
ZGCDS exerts neuroprotective effects by inhibiting the activation of TNF/NF-κB and Ras/ERK signaling pathways in SH-SY5Y cells (A) WB was used to detect the representative expressions of TNFR1, P65, and p-P65 in SH-SY5Y cells. (B) Statistics of the relative expression level of TNFR1 in SH-SY5Y cells (*n* = 3). (C) Statistics of the relative expression level of p-P65/P65 in SH-SY5Y cells (*n* = 3). (D–G) IF was used to analyze the expressions of TNFR1 and p-P65 in SH-SY5Y cells (*n* = 3). Scale bars, 200 μm. (H–J) WB analysis of Ras and ERK expression and phosphorylation in SH-SY5Y cells (*n* = 6). (K–P) IF was used to analyze the expressions of Grb2, Ras, and *p*-ERK in SH-SY5Y cells (*n* = 3). Scale bars, 100 μm. (Q–S) WB analysis of TNFR1 and P65 expression and phosphorylation in SH-SY5Y cells (*n* = 4). (T–W) WB analysis of Grb2, Ras, and ERK expression and phosphorylation in SH-SY5Y cells (*n* = 4). ###*p* < 0.001 vs. Con; n.s., not significant, ∗*p* < 0.05, ∗∗*p* < 0.01 and ∗∗∗*p* < 0.001 vs. MPTP. Data are expressed as mean ± SD.

In order to comprehensively evaluate the regulatory effect of ZGCDS on the TNF/NF-κB and Ras/ERK signaling pathways *in vitro*, we adopted a comprehensive method including WB, IF, and RT-qPCR to assess the expression levels of pathway proteins and genes. Experimental results demonstrated marked activation of both TNF/NF-κB and Ras/ERK signaling pathways in MPP^+^-exposed cells. WB analysis demonstrated significantly elevated protein levels of TNFR1 and p-P65 in MPP^+^-treated cells, whereas ZGCDS intervention substantially suppressed these increases (Figures 8A–8C). IF confirmed intensified TNFR1 fluorescence intensity and nuclear translocation of p-P65 in MPP^+^-exposed cells, pathological alterations that were effectively reversed by ZGCDS treatment (Figures 8D–8G). Moreover, RT-qPCR analysis corroborated these findings at the transcriptional level, showing concordant downregulation of *Tnfr1* and *nfkb* expression following ZGCDS administration (Figures S1D and S1E). Concurrently, integrated analyses via WB, IF, and RT-qPCR demonstrated that MPP^+^ exposure upregulated Ras and *p*-ERK expression while elevating Grb2—an upstream adaptor protein regulating Ras activation. ZGCDS treatment significantly attenuated the expression of these effectors, achieving efficacy comparable to L-DOPA intervention (Figures 8H–8P; Figures S1F–S1H). These findings demonstrate that ZGCDS likely confers neuroprotection by modulating TNF/NF-κB and Ras/ERK signaling pathways, thereby attenuating MPP^+^-induced cellular damage through the suppression of neuroinflammatory cascades and mitochondrial apoptotic pathways. R-7050 is a small molecule that inhibits TNFR1 signaling by blocking its interaction with intracellular adaptor proteins. This inhibition prevents receptor complex internalization and downstream signaling, thereby effectively suppressing the activation of key pro-inflammatory pathways such as NF-κB.^22^ To further investigate the potential relationship between the two pathways, we employed the TNFR1 inhibitor R-7050 to suppress the activation of the TNF/NF-κB pathway. We referred to the dosage in previous literature and pretreated SY5Y cells with 10 μM R-7050.^23^ We observed that treatment with R-7050 effectively blocked the activation of both TNFR1 and NF-κB, confirming the successful inhibition of the TNF/NF-κB signaling cascade (Figures 8Q–8S). We next examined the expression of downstream components Grb2 and Ras. Results showed that even after TNF/NF-κB pathway inhibition, MPP^+^ treatment still significantly up-regulated Grb2 and Ras expression. Moreover, administration of ZGCDS effectively suppressed this up-regulation. These findings suggest that the TNF/NF-κB pathway does not appear to directly activate Grb2 and Ras expression. However, when assessing ERK phosphorylation, we found that the inhibition of the TNF/NF-κB pathway partially suppressed the MPP^+^-induced phosphorylation of ERK (Figures 8T–8W). Under this condition, ZGCDS treatment still conferred a certain degree of therapeutic effect, although this effect was notably weaker than that observed in MPP^+^-treated cells without the inhibitor. This indicates that the activation of the TNF/NF-κB pathway may contribute to the enhancement of ERK phosphorylation to some extent. Collectively, these findings indicate that while the two pathways can function independently, they also exhibit synergistic crosstalk.

### Bioactive compounds from Zhigancao Decoction may exert anti-Parkinson’s disease effects by targeting the TNF/NF-kB and Ras/ERK pathways

**Figure 9:**
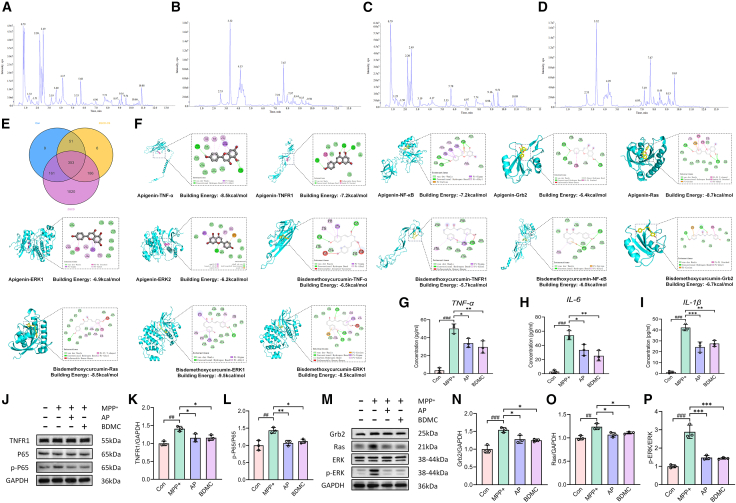
Bioactive compounds (Apigenin, Bisdemethoxycurcumin) from ZGCD may exert anti-PD effects by targeting the TNF/NF-kB and Ras/ERK pathways (A and B) Total ion chromatograms (TICs) of Con in positive (A) and negative (B) ion modes. (C and D) Total ion chromatograms (TICs) of ZGCD-CS in positive (C) and negative (D) ion modes. (E) Venn diagram of serum pharmacochemical analysis. (F) Molecular docking analysis of Apigenin and Bisdemethoxycurcumin with key proteins in the pathway. (G–I) The levels of TNF-α, IL-1β, and IL-6 were detected by ELISA in SH-SY5Y cells (*n* = 3). (J–L) WB analysis of TNFR1 and P65 expression and phosphorylation in SH-SY5Y cells (*n* = 3). (M–P) WB analysis of Grb2, Ras, and ERK expression and phosphorylation in SH-SY5Y cells (*n* = 3). ###*p* < 0.001 vs. Con; n.s., not significant, ∗*p* < 0.05, ∗∗*p* < 0.01 and ∗∗∗*p* < 0.001 vs. MPTP. Data are expressed as mean ± SD.

Due to the complex composition of TCM, we concurrently characterized chemical constituents in ZGCD-containing serum (Figures 9A and 9B) and normal serum (Figures 9C and 9D) to identify potential anti-PD bioactive compounds. Table S3 provides complete details of all identified substances. The 186 shared compounds between ZGCD-CS and ZGCD represent the bioavailable fraction retained after *in vivo* absorption, forming the pharmacological basis of ZGCD’s efficacy, such as Apigenin (AP), Nornuciferine, Aeginetic acid, Zizybeoside II, Ginsenoside Rh4, Vitexin, and Luteolin, which are consistent with the components screened by network pharmacology. AP exhibits a variety of biological effects in alleviating neurodegeneration, including antioxidant, anti-inflammatory, and anti-apoptotic activities.^24^ Multiple studies have shown that AP can improve motor dysfunction and apoptosis in PD models by inhibiting neuroinflammation and oxidative stress.^25^^,^^26^ Furthermore, six unique compounds in ZGCD-CS likely constitute metabolites generated through biotransformation, such as Bisdemethoxycurcumin (BDMC), Arcapillin, Soyasapogenol C, and 11-Keto-ursolic acid. These distinctive metabolites may exhibit significant bioactivity and potentially serve as critical mediators of ZGCD’s neuroprotective effects. Among them, the content of dimethoxy curcumin is high. Studies have shown that BDMC has strong antioxidant, anti-inflammatory, and anti-apoptotic activities, and can effectively prevent and alleviate neurodegenerative diseases.^27^ It is a drug with clinical application prospects. Multiple studies have shown that BDMC can improve the symptoms of Parkinson’s disease by reducing oxidative stress and neuroinflammation.^28^^,^^29^ Therefore, we took AP and BDMC as representative compounds to explore the mechanism of action of active substances in ZGCD on PD. First, we conducted molecular docking to analyze the interactions between these bioactive substances and key pathway targets. AP and BDMC were molecularly conjugated with TNF-α, TNFR1, NF-κB, Grb2, Ras, ERK1 and ERK2. Both ligands exhibited binding affinities < −5.0 kcal/mol to key targets along TNF/NF-κB and Ras/ERK pathways (Table 3). Their interactions involved multiple binding modalities, such as hydrogen bonding, carbon-hydrogen bonding, and π-π stacking (Figure 9F). Molecular docking prediction shows that apigenin has a strong binding affinity for TNF-α and NF-κB, while BDMC has a stronger binding ability for Ras and ERK. This indicates that different components of ZGCD can simultaneously target and inhibit multiple different pathogenic pathways, forming a broad-spectrum inhibitory network. Furthermore, the docking also indicates that these two compounds can bind to different proteins in the same primary link. This means that they can synergistically suppress signal transduction at multiple steps within a single pathway, thereby resulting in a more powerful and effective blocking than targeting a single node. We further verified the results through *in vitro* experiments. Based on previous literature establishing a safe concentration range of 2.5–20 μM for AP in SH-SY5Y cells, we first treated MPP^+^-injured cells with varying AP concentrations.^30^ The results indicated that 20 μM AP produced the optimal therapeutic effect (Figure S1R). Subsequently, we evaluated different concentrations (2.5–30 μM) of BDMC and observed cytotoxic effects at 20 μM, while concentrations between 2.5 and 15 μM showed no significant cytotoxicity (Figure S1S). Further testing within the 2.5–15 μM range revealed that 15 μM BDMC provided the most substantial therapeutic benefit in MPP^+^-treated cells (Figure S1T). Consequently, subsequent experiments were conducted using 20 μM AP and 15 μM BDMC. We used ELISA to detect the effects of these two compounds on inflammatory factors in SY5Y cells induced by MPP^+^. We found that both of these compounds could effectively reduce the inflammatory response (Figures 9G–9I). Then, we respectively detected the regulatory effects of the two compounds on the TNF/NF-κB and Ras/ERK pathways through WB. The results indicated that both compounds could effectively inhibit the activation of the two pathways (Figures 9J–9P). These findings suggest ZGCD’s chemical constituents may inhibit pathway activation by interacting with critical targets in TNF/NF-κB and Ras/ERK cascades. The receptor-ligand binding in ZGCD’s anti-PD action is mediated not by single chemical bonds but through complex polyvalent interactions, maintaining stable binding conformations. This exemplifies the multi-component, multi-target mechanisms characteristic of TCM. It provides a basis for further exploration of the mechanism by which ZGCD alleviates PD in the future.

**Table 3:** Docking effect analysis of compounds and targets

Compound	Target	Binding energy (kcal/mol)
Apigenin	TNF-α	-6.9
Apigenin	TNFR1	-6.2
Apigenin	NF-κB	-7.2
Apigenin	Grb2	-6.4
Apigenin	Ras	-8.7
Apigenin	ERK1	-8.5
Apigenin	ERK2	-7.2
Bisdemethoxycurcumin	TNF-α	-6.4
Bisdemethoxycurcumin	TNFR1	-6.4
Bisdemethoxycurcumin	NF-κB	-6.7
Bisdemethoxycurcumin	Grb2	-6.7
Bisdemethoxycurcumin	Ras	-8.5
Bisdemethoxycurcumin	ERK1	-8.0
Bisdemethoxycurcumin	ERK2	-7.6

## Discussion

PD is a prevalent neurodegenerative disorder characterized by complex, multifactorial pathogenesis. This pathological complexity presents a persistent clinical challenge for developing long-term effective therapies. Our findings demonstrate that ZGCD exerts pronounced neuroprotective effects, effectively alleviating motor deficits in murine PD models, thereby providing a promising therapeutic avenue for PD management. Pharmacological studies have also shown that various bioactive components in ZGCD exhibit significant DA neuroprotective properties and have therapeutic potential for neurodegenerative diseases. The etiology of PD remains unclear at present, involving the regulation of multiple factors, which limits the therapeutic effect of drugs on patients with PD.^31^ Neuroinflammation and apoptosis play important roles in PD. Most TCM compounds alleviate apoptosis and slow down the progression of the disease through anti-inflammatory and antioxidant stress.^32^^,^^33^ This study provides a basis for the further exploration of the pathogenesis and treatment of PD.

Bioinformatics is an important approach for exploring therapeutic targets and mechanisms of diseases and developing innovative drugs.^34^ ZGCD’s phytochemical constituents enable multi-target modulation, characteristic of traditional herbal polypharmacology, which likely mediates its anti-PD effects. Consequently, we leveraged network pharmacology to predict principal therapeutic targets for PD intervention by ZGCD. Our methodology identified 736 candidate compounds via the TCMSP and HERB database, with subsequent target mapping revealing 95 hub targets intersecting with established PD-associated pathways. Building upon these findings, GO and KEGG enrichment analyses established mechanistic connections between ZGCD’s neuroprotective actions and coordinated regulation of TNF/NF-κB and Ras/ERK signaling cascades, effectively mitigating neuroinflammatory processes and apoptotic dysregulation.

We established PD models exhibiting characteristic motor deficits through the intraperitoneal administration of MPTP. This neurotoxin is extensively employed to induce PD pathology in C57BL/6 mice.^35^ Behavioral assessments demonstrated that ZGCD significantly ameliorated MPTP-induced motor dysfunction *in vivo*. IHC and IF co-analyses confirmed ZGCD treatment prevented MPTP-induced loss of TH^+^ neurons in the SNpc. WB quantification further confirmed that ZGCD upregulated nigral TH protein expression while downregulating pathological α-syn accumulation. Licorice and Rehmanniae Radix constitute the principal constituents of ZGCD. Substantial preclinical evidence confirms the neuroprotective efficacy of these bioactive compounds and standardized botanical extracts. These well-documented properties establish such botanicals as validated neuroprotective agents within traditional medicine paradigms for neurodegenerative disease management.^36^^,^^37^ Matteo et al. reported that glycyrrhizin dose-dependently protects TH^+^ neuronal populations in the SN of MPTP-intoxicated mice. This intervention significantly attenuated DA cell death.^38^ A clinical trial further demonstrated that supplementation with polyphenol-enriched licorice extract significantly improves Unified Parkinson’s Disease Rating Scale total scores as an adjunct therapy for patients with PD, ameliorating activities of daily living and tremor severity.^39^ Catalpol enhances motor function by attenuating neurodegeneration along the nigrostriatal DA pathway. As the principal iridoid glycoside in Rehmanniae Radix, this compound demonstrates significant neuroprotective efficacy.^40^ Collectively, these findings substantiate that ZGCD effectively restores DA neuronal integrity and confers significant therapeutic efficacy against PD motor symptomatology.

Neuroinflammation and apoptosis constitute pivotal pathological mechanisms in PD pathogenesis.^41^ Accumulating evidence has demonstrated that MPTP-induced PD murine models exhibit robust neuroinflammatory responses characterized by elevated pro-inflammatory cytokines (TNF-α, IL-1β, IL-6) and consequent neuronal apoptosis within the nigrostriatal circuitry.^42^^,^^43^ Sustained activation of glial cells triggers inflammatory cascades, releasing cytotoxic cytokines including TNF-α, IL-1β, and IL-6. These mediators exacerbate neuronal injury by potentiating mitochondrial apoptotic pathways and oxidative stress, ultimately accelerating neurodegeneration and disease progression through DA synapse dysfunction.^44^ In this investigation, we demonstrate that ZGCD and ZGCDS significantly reduce pro-inflammatory cytokine levels (TNF-α, IL-1β, and IL-6) in both MPTP-intoxicated murine brain tissue and MPP^+^-treated SH-SY5Y cells. Furthermore, experimental analyses revealed that ZGCD and ZGCDS treatment suppressed the expression of apoptotic biomarkers, including Bax and Cleaved Caspase-3, while upregulating the anti-apoptotic protein Bcl-2. These findings establish that ZGCD exerts potent anti-inflammatory and anti-apoptotic properties across *in vivo* and *in vitro* systems, effectively mitigating neuroinflammation and apoptosis-mediated DA neuronal loss.

The TNF/NF-κB and Ras/ERK signaling pathways critically regulate inflammatory cascades and cellular survival/death decisions. TNF-α acts as a primary inflammatory driver by specifically binding and activating TNFR1 receptors, which subsequently initiate NF-κB nuclear translocation to amplify pro-inflammatory cytokine production.^45^ Context-dependently, TNFR1 engagement may alternatively trigger apoptotic cascades. Studies have shown that the activation of the Ras/ERK pathway in the MPP^+^ cell model leads to the death of neuronal cells. The activation of ERK is bidirectionally regulated—its physiological consequences are determined by activation kinetics (duration/magnitude) and subcellular compartmentalization.^46^ A study in 1996 found that the activation of the Ras/ERK pathway promoted the generation of apoptosis.^47^ In PD models, aberrant ERK activation correlates pathologically with neuronal hyperexcitability and cell death. Wang et al. demonstrated that MPTP-induced PD mice exhibit co-activated ERK/JNK pathways driving neuroinflammation and neuronal apoptosis, with cordycepin intervention reversing these pathologies through targeted pathway inhibition.^48^ Our investigation demonstrated that MPTP/MPP^+^-induced PD models in both the murine model and SH-SY5Y cells exhibited TNF/NF-κB and Ras/ERK signaling hyperactivation. This dysregulation featured elevated TNF-α levels, TNFR1 upregulation, NF-κB nuclear translocation, and enhanced Ras/ERK phosphorylation. Furthermore, our investigation into the potential crosstalk between the two pathways revealed that the activation of the TNF/NF-κB pathway appears to contribute to the enhancement of ERK phosphorylation, without affecting the upstream regulators Grb2 and Ras. This indicates that while the two pathways largely function independently, a partial synergistic interaction exists between them.

The chemical components in ZGCD decoction, ZGCD-containing serum, and normal serum were identified by UPLC-MS/MS. A total of 1720 compounds were identified in the ZGCD decoction, involving various components such as flavonoids, phenolic acids, terpenoids, and alkaloids. In addition, by combining the components in the drug-containing serum and the normal serum, we can focus on the 186 active substances shared by the ZGCD decoction and the drug-containing serum, as well as the 6 active substances unique to the drug-containing serum. These may constitute the pharmacological basis of ZGCD’s anti-PD effects and may serve as key mediators of its neuroprotection. Evidence based on serum drug chemistry analysis indicates that active compounds such as Apigenin and Bisdemethoxycurcumin can be absorbed into the systemic circulation via the oral route, serving as the potential pharmacological substance basis with certain bioavailability. Apigenin is becoming increasingly important as a health enhancer. For a long time, it has been regarded as safe, with low toxicity levels and seemingly capable of crossing the blood-brain barrier.^49^ The delayed plasma clearance and slow decomposition of Apigenin in the liver increase its systemic bioavailability, making AP an effective therapeutic agent in drug development. However, its poor water solubility and limited membrane permeability still limit its clinical application.^50^ A study has shown that apigenin, when coupled with amino acids, significantly enhances its solubility and ability to cross the blood-brain barrier, and ideally perturbs the central nervous system, providing information for clinical use.^51^ Bisdemethoxycurcumin is a derivative of curcumin. Curcumin is a well-known phenolic compound extracted from turmeric, which has a variety of beneficial properties, including antioxidant, anti-inflammatory, and neuroprotective properties. However, it has low water solubility, minimal polarity, is prone to degradation in alkaline environments, and faces challenges in crossing the blood-brain barrier.^52^ Compared with its parent compound, Bisdemethoxycurcumin exhibits higher polarity, water solubility and metabolic stability, and is one of the candidate drugs for the clinical remission of neurological diseases.^53^ Therefore, directly determining the dynamic concentration changes of these components in brain tissue through more advanced technologies will be the most valuable deepening direction in future research, providing more direct information for the development and clinical application of drugs. Molecular docking is an indispensable tool in drug discovery and molecular modeling applications.^54^ Our molecular docking studies have shown that key active substances such as Apigenin and Bisdemethoxycurcumin exhibit strong interactions with key targets in the TNF/NF-κB and Ras/ERK pathways and have multiple binding modes. And through *in vitro* experiments, it was verified that they could inhibit the abnormal activation of the TNF/NF-κB and Ras/ERK pathways. The two active substances can jointly alleviate inflammation and synergistically inhibit the activation of pathways. Therefore, these results suggest that the active substances contained in ZGCD may inhibit the excessive activation of the TNF/NF-κB and Ras/ERK pathways by binding to multiple targets on them.

### Limitations of the study

However, the multi-component and multi-target nature of ZGCD also represents a limitation of this study. This complex mechanism of action poses a challenge in elucidating its precise molecular basis, as we have only validated two of its active constituents. Therefore, in future research, we will systematically investigate the mechanisms of other active substances in ZGCD, thereby gaining a more comprehensive understanding of the principles underlying its neuroprotective effects.

### Conclusions

Collectively, this study demonstrates that ZGCD exerts neuroprotective effects in MPTP-induced PD mice and MPP^+^-intoxicated SH-SY5Y cells. ZGCD ameliorates motor deficits in PD murine models and attenuates neuroinflammation and apoptosis through: inhibition of TNF/NF-κB and Ras/ERK signaling cascades, reduction of pro-inflammatory cytokine release (TNF-α, IL-1β, IL-6), and transcriptional and translational regulation of apoptotic regulators. The multiple active substances in ZGCD interact through various molecules, influencing the cascade reactions of the TNF/NF-κB and Ras/ERK pathways, and reducing the degeneration of the SN and striatum to protect DA neurons. These results indicate the potential of ZGCD as a promising therapeutic agent in the treatment of PD.

## Resource availability

### Lead contact

Further information and requests for resources and reagents should be directed to and will be fulfilled by the lead contact, Yan Lu (sophieluo896@njucm.edu.cn).

### Materials availability

This study did not generate new unique reagents.

### Data and code availability

• Data: Data generated in this study and reported in this article will be shared by Dr. Yan Lu (sophieluo896@njucm.edu.cn) upon request.
• Code: This article does not report original code.
• Additional Information: Any additional information required to reanalyze the data reported in this article is available from the lead contact upon request.

## Acknowledgments

The author expresses gratitude for the financial support from the 10.13039/501100001809National Natural Science Foundation of China (Project number: 81804022) and Jiangsu Province Postgraduate Practical Innovation Project (Project Number: SJCX24_1121).

## Author contributions

J.Z., X.Y., Y.F., and Y.L. designed this research. J.Z., X.Y., Y.F., and W.L. performed the experiments. Q.C., X.L., Y.Z., and Y.J. helped with the data analysis. J.Z., X.Y., Y.F., and C.X. wrote the article. Y.L. and X.S. supervised the study and revised the article. All authors read and approved the final article. All authors agree to be responsible for all aspects of the work to ensure completeness and accuracy.

## Declaration of interests

The authors declare no competing interests.

## STAR★Methods

### Key resources table

**Table:** 

REAGENT or RESOURCE	SOURCE	IDENTIFIER
**Antibodies**		

Rabbit monoclonal to TH	Abcam	Cat#ab137869; RRID: AB_2801410
Rabbit monoclonal to α-Syn	Abcam	Cat#ab138501; RRID: AB_2537217
Rabbit monoclonal to Ras	Abcam	Cat#ab52939; RRID: AB_2121042
Rabbit monoclonal to Grb2	Abcam	Cat#ab32111; RRID: AB_2113026
Rabbit polyclonal to ERK	Proteintech	Cat#11257-1-AP; RRID: AB_2139822
Rabbit polyclonal to *p*-ERK	Proteintech	Cat#28733-1-AP; RRID: AB_2881202
Rabbit polyclonal to TNF-α	Proteintech	Cat#17590-1-AP; RRID: AB_2271853
Rabbit polyclonal to TNFR1	Proteintech	Cat#21574-1-AP; RRID: AB_10734433
Rabbit monoclonal to P65	Abcam	Cat#ab32536; RRID: AB_776751
Rabbit monoclonal to p-P65	Abcam	Cat#ab76302; RRID: AB_1524028
Rabbit polyclonal to Bax	Proteintech	Cat#50599-2-Ig; RRID: AB_2061561
Rabbit polyclonal to Bcl-2	Proteintech	Cat#26593-1-AP; RRID: AB_2818996
Rabbit polyclonal to Caspase-3	Proteintech	Cat#19677-1-AP; RRID: AB_10733244
Rabbit polyclonal to Cleaved-Caspase-3	Proteintech	Cat#25128-1-AP; RRID: AB_3073913
Rabbit monoclonal to GAPDH	Abcam	Cat#ab181602; RRID: AB_2630358
Anti-rabbit IgG, HRP-linked Antibody	Proteintech	Cat#SA00001-2; RRID: AB_2722564
Anti-rabbit IgG, AF488 antibodies	Invitrogen	Cat#A-11094; RRID: AB_221544

**Chemicals, peptides, and recombinant proteins**		

MPTP	MCE	Cat#HY-15608
L-DOPA	MCE	Cat#HY-N0304
MPP+	MCE	Cat#HY-W008719
Apigenin	Macklin	Cat#A800500
Bisdemethoxycurcumin	Sigma	Cat#33171-05-0
R-7050	MCE	Cat#HY-110203

**Critical commercial assays**		

CCK-8 Cell Counting Kit	Vazyme	Cat#A311-01
BCA assay kit	Beyotime	Cat#P0012
FastPure Cell/Tissue Total RNA Isolation Kit V2	Vazyme	Cat#RC112-01

**Experimental models: Cell lines**		

SH-SY5Y	ATCC	CRL-2266

**Experimental models: Organisms/strains**		

male SD rats	Beijing Weitahe Experimental Animal Technology Co.	N/A
Male C57BL/6 mice	Shanghai SLAC Laboratory Animal Co.	N/A

**Oligonucleotides**		

Primers for the study, Please see Table S1	N/A	N/A

**Software and algorithms**		

GraphPad Prism	GraphPad Inc.	https://www.graphpad.com
ImageJ	NIH	https://imagej.nih.gov/ij/
R (v4.3.1 or later)	The R Foundation	https://www.r-project.org/
Cytoscape software	N/A	https://cytoscape.org/

### Experimental model and study participant details

#### Ethics approval

This study was approved by the Animal Ethics Committee of Nanjing University of Chinese Medicine (Approval No.: A20241201) and conducted in accordance with institutional guidelines.

#### Animal model

Male C57BL/6 mice (8–10 weeks, 20–22 g) were procured from Shanghai SLAC Laboratory Animal Co. and maintained in specific pathogen-free facilities at Nanjing University of Chinese Medicine’s Animal Center. Housing conditions included a temperature-controlled environment (22 ± 2°C) with 50 ± 5% relative humidity and a standardized 12-h light/dark cycle. Animals received *ad libitum* access to food and water. The MPTP-induced PD model reproduces the pathological features of PD at multiple levels. The neurotoxicity induced by injection has been shown to produce both motor and non-motor symptoms of PD in animal models, with no severe hypersensitivity side effects observed.^55^ Therefore, MPTP is an ideal choice for modeling PD. All experimental protocols were approved by the Institutional Animal Ethics Committee at Nanjing University of Chinese Medicine (Approval No. A20241201) and conducted in compliance with institutional guidelines. According to the body surface area equivalent dose conversion principle, the medium dose of ZGCD was determined to be 17.81 g/kg/d. The low-dose group (8.91 g/kg/d) and high-dose group (35.62 g/kg/d) were set to half and double the medium dose, respectively.^56^ Following a 7-day acclimatization period, mice were randomly allocated into six experimental groups (*n* = 8/group): Control group (Con): Received daily intraperitoneal (i.p.) saline injections for 7 days and saline gavage for 4 weeks. MPTP model group: Administered i.p. MPTP (30 mg/kg/day) for 7 days with concurrent saline gavage for 4 weeks. ZGCD low-dose group (ZGCDL): Treated with i.p. MPTP (30 mg/kg/day) and ZGCD (9 g/kg/day) gavage for 4 weeks. ZGCD medium-dose group (ZGCDM): Received i.p. MPTP (30 mg/kg/day) and ZGCD (18 g/kg/day) gavage. ZGCD high-dose group (ZGCDH): Administered i.p. MPTP (30 mg/kg/day) and ZGCD (36 g/kg/day) gavage. Positive control group (L-DOPA): Treated with i.p. MPTP (30 mg/kg/day) and L-DOPA (20 mg/kg/day) gavage. All intervention measures were initiated simultaneously with the induction of MPTP injection and all administration frequencies are once a day.

#### Cell culture

The human neuroblastoma cell line SH-SY5Y was obtained from the American Type Culture Collection (ATCC, USA). To induce *in vitro* neurotoxicity, SH-SY5Y cells were exposed to 1-methyl-4-phenylpyridinium (MPP^+^; MCE, China, HY-W008719, 99.94%). The cells were maintained in high-glucose Dulbecco’s Modified Eagle’s Medium supplemented with 10% fetal bovine serum, 100 IU/mL penicillin and 100 μg/mL streptomycin (DMEM; Gibco, Thermo Fisher Scientific, USA) supplemented with 10% fetal bovine serum (FBS; Gibco) and cultured in a humidified incubator at 37°C under 5% CO_2_ and 95% air. Cells at the exponential growth phase were harvested for subsequent experiments.

### Method details

#### Network pharmacological study on the effect and mechanism of Zhigancao decoction in treating Parkinson’s disease

##### Collection of drug active ingredient targets

The active ingredients of individual herbal components in ZGCD were systematically retrieved from the TCM Systems Pharmacology Database (TCMSP: https://old.tcmsp-e.com/tcmsp.php) and the HERB database (HERB database: http://herb.ac.cn/). Active compounds were screened based on oral bioavailability (≥30%) and drug-likeness (≥0.18). Canonical SMILES structures of these bioactive compounds were acquired via the PubChem database (PubChem database: https://pubchem.ncbi.nlm.nih.gov/). The resulting UniProt IDs of predicted target proteins were converted to standardized Gene Symbols using UniProt’s ID mapping tool. Duplicate entries were removed to generate a non-redundant list of bioactive compound-target interactions.

#### Acquisition of disease-related targets

Using “Parkinson’s disease” as a keyword, we screened for PD-related genes from the GeneCards, DrugBank, Comparative Toxicogenomics Database, DisGeNET, PharmGKB and Online Mendelian Inheritance in Man. Initial target datasets from these repositories were consolidated, and duplicate entries were rigorously removed. Subsequently, the remaining targets were standardized using the UniProt database to ensure nomenclature consistency and functional annotation accuracy, yielding a non-redundant set of PD-associated molecular targets.

#### Venn diagram drawing and drug component target network drawing

Potential therapeutic targets mediating the pharmacological effects of the herbal components were identified by intersecting compound-target profiles with disease-associated targets using the Venny online analytical platform (v2.1.0). This integrative approach generated a consensus target set representing putative molecular nodes for disease intervention. Finally, a multi-layered “herb-component-core target-disease” interaction network was constructed and subjected to topological parameter analysis using Cytoscape software (v3.9.1).

#### Protein-protein interaction (PPI) network mapping

The cross-targets of the complex disease obtained above were imported into the STRING database (STRING database: https://cn.string-db.org/), with Homo sapiens defined as species and the cut-off score of the interaction score >0.900. Analyze and construct the target PPI network.

#### GO and KEGG enrichment analysis

The critical targets identified above were subjected to Gene Ontology (GO) and Kyoto Encyclopedia of Genes and Genomes (KEGG) pathway enrichment analyses using the Metascape platform (Metascape platform: https://metascape.org/), with species restricted to Homo sapiens and parameters maintained at default settings. Biological Process (BP), Molecular Function (MF), and Cellular Component (CC) terms were filtered based on Log *p* values, retaining the top 10 enriched terms in each GO category. Similarly, the top 20 significantly enriched KEGG pathways were selected. The filtered GO and KEGG results were imported into R software (v4.3.1), where the ggplot2 package (v3.4.2) was employed to generate a horizontal bar plot for GO term visualization and a bubble plot for KEGG pathway representation.

#### Preparation of the ZGCD

ZGCD is composed of 9 kinds of TCM. The detailed information such as the names, dosages, batch numbers and origins of these drugs is shown in Table 1. It is purchased from the Pharmacy Department of Nanjing Hospital of Traditional Chinese Medicine. And it complies with the standards outlined in the 2020 Edition of the Pharmacopoeia of the People’s Republic of China. Soak the herbs in water for 30 min, then boil them over high heat and simmer them over low heat for 60 min. Then filter the decoction to remove the residue. After three rounds of decoction and reflux extraction, the combined extract was filtered using a rotary evaporator and concentrated to 2.5 g/mL and refrigerate for later use. To ensure the consistency of the decoction process, the weight and decoction time of all herbs are strictly followed in accordance with the standard operating procedures. The volume and concentration of each decoction are kept consistent.

**Table 1:** The compositions of ZGCD

Latin name	Chinese name	Medicinal part	Batch number	Daily adult dose (g)	Origin
Rehmannia glutinosa	Shengdihuang	Root	230902	50	Henan, China
Glycyrrhiza uralensis Fisch.	Zhigancao	Rhizome and Root	2306003	12	Gansu, China
Panax ginseng	Renshen	Rhizome and Root	230801	6	Jilin, China
Ziziphus Jujuba Mill.	Dazao	Dry fructus	230901	25	Xinjiang, China
Asini Corii Colla	Ejiao	Dry skin	2209171	6	Hubei, China
Ophiopogonis Radix	Maidong	Tuberous Root	A231010	10	Anhui, China
Cinnamomi Ramulus	Guizhi	twig	A231018	9	Anhui, China
Zingiber officinale Roscoe	Shengjiang	Rhizome and Root	230903	9	Anhui, China
Cannabis sativa L.	Maren	fructus	230901	10	Anhui, China

#### Preparation of the drug-containing serum

Serum containing ZGCD drugs (ZGCDS) was prepared by the serum pharmacology method. Twenty healthy 8-week-old male SD rats (200 ± 20 g) were purchased from Beijing Weitahe Experimental Animal Technology Co., Ltd. and were divided into two groups, with 10 rats in each group. According to the principle of equivalent dose conversion based on body surface area and in combination with the standard administration norms for experimental animals, one group was intragastric with a dose of 12 g/kg/d for 7 consecutive days.^56^ The control group received the same volume of distilled water through tube feeding at the same time every day for 7 consecutive days. 2 h after the final treatment, abdominal aortic blood was collected under sterile conditions and centrifuged at 3000 rpm for 15 min to separate the serum containing the ZGCD drug. Another group was gavage with normal saline to obtain blank serum. All serum samples were passed through a 0.22 μm filter, inactivated in a water bath at 56°C for 30 min, and stored at −20°C until use.

#### UPLC-MS/MS analysis

The chemical components in ZGCD, ZGCD-containing serum (ZGCD-CS) and normal serum (Con) were identified by UPLC-MS/MS. Liquid chromatography conditions: Chromatographic column: Agilent SB-C18, 1.8 μm, 2.1 mm × 100 mm. Mobile phase: A: Ultrapure water containing 0.1% formic acid. Acetonitrile with 0.1% formic acid. Gradient elution: 0.00 min, with 5% in phase B. From 0.00 to 9.00 min, phase B increased linearly to 95%. Maintain at 95% for 1 min. From 10.00 to 11.10 min, phase B decreased to 5%. Balance within 5%–14 min. Flow rate: 0.35 mL/min. Column temperature: 40°C. The mass spectrometry conditions mainly include: electrospray ionization at a temperature of 500°C; Ion spray voltage: 5500 V (positive ion mode)/-4500 V (negative ion mode); The ion source gas I (GSI), gas II (GSII) and gas curtain gas (CUR) were set at 50, 60 and 25 psi respectively, and the collision-induced ionization parameters were set to high. The QQQ scan uses the MRM mode and sets the collision gas (nitrogen) to medium. Through further optimization of the declustering potential (DP) and collision energy (CE), the DP and CE of each MRM ion pair were completed. Based on the metabolites eluted in each period, a specific set of MRM ion pairs was monitored in each period.

#### Cell viability assay

Cells (5 × 10^4^/well) were exposed to different concentrations of MPP^+^, ZGCDS, Apigenin (AP, A800500, Macklin, China), or Bisdemethoxycurcumin (BDMC, Cat# 33171-05-0, Sigma-Aldrich, St. Louis, MO, USA) for different durations. ZGCDS (5%, 10%, 15%, 20%, 25%, 30%, 40%, 50%) were detected for 48 h, and MPP^+^ (0–2 mM), AP (2.5–20 μM) or BDMC (2.5–30 μM) were detected for 24 h. After treatment, 10 μL of CCK-8 solution was added to each well, incubated at 37° C for 1 h, and the absorbance was measured at 450 nm.

#### Behavioral tests

##### Open field test

An open-field apparatus (45 × 45 × 60 cm) was employed for behavioral testing. After a 30-min habituation period, mice were positioned in the arena center, with automated tracking (EthoVision XT) immediately recording locomotion distance and velocity. The central zone was defined as a square area occupying 25% of the total arena floor area (i.e., 22.5 × 22.5 cm, located in the center of the arena). The remaining area was designated as the peripheral zone. Inter-session sanitization involved 75% ethanol disinfection and complete air-drying.

##### Gait analysis

The CatWalk system served as an automated gait analysis platform for quantitative assessment of motor function and interlimb coordination in freely moving mice. System calibration required presetting camera-to-platform distance and software parameters, with locomotion trials lasting <10 s deemed valid. Mice underwent three consecutive days of apparatus habituation, and ≥5 uninterrupted crossings per subject were recorded. Experimental sessions occurred in a sound-attenuated, dimly lit environment, with walkway disinfection using 70% ethanol between trials to eliminate olfactory cues.

##### Rotarod test

For rotarod testing, mice were placed on an accelerating rotating rod and the rotation speed started at 4 revolutions per min (rpm) and increased linearly to 40 rpm over a 5-min period. Following three days of habituation, formal trials recorded latency-to-fall (seconds) as the duration of balance maintenance. Each subject underwent ≥3 trials with ≥30-min inter-session intervals, with mean latency calculated across trials.

##### Pole test

A wooden pole (16 mm diameter, 60 cm height) was utilized for testing. Each mouse was positioned head-up at the pole’s apex, and descent latency (time to base) was recorded. Five trials per mouse were conducted, with the mean descent time calculated. All animals underwent three days of pre-training prior to testing.

##### Forced swimming test (FST)

The behavioral desperation of the experimental mice was evaluated by the FST. Under the condition of 25 ± 1°C, each mouse was placed separately in a glass bottle with a diameter of 20 cm and a height of 50 cm, filled with 30 cm deep tap water to prevent tail bronchitis. The test record lasts for 6 min, with the first 2 min being the pre-test adaptation period. The total rest time of the last 4 min was quantified. After the experiment, each mouse was gently dried with a paper towel, placed under a heating lamp to restore its body temperature, and then returned to its home cage. After each experiment, the water should be changed to eliminate potential confusing olfactory cues and maintain consistency throughout the experiment. Immobility time is defined as the period during which a rat’s head emerges from the water and its limbs move only slightly. An increase in immobility time indicates an increase in depressive-like behavior.

##### Elevated plus maze test (EPM)

EPM is a widely used test for assessing anxiety-related behaviors in rodents. The apparatus consists of four arms: two open arms and two enclosed arms, each measuring 50 cm in length and 10 cm in width. The enclosed arms feature 40 cm high walls, while the open arms have no walls. All arms are connected by a central platform (10 × 10 cm), with the entire maze elevated 80 cm above the floor. At the beginning of each trial, a mouse was placed on the central platform facing an open arm and allowed to freely explore the maze for 5 min. The time spent in the open arms was quantified using a high-resolution infrared camera and tracking software. After each test session, the maze was thoroughly wiped with 75% ethanol to eliminate residual odors.^57^

#### Immunofluorescence (IF)

Mice were deeply anesthetized with isoflurane and transcardially perfused with 10 mL PBS (0.9%), followed by 30 mL paraformaldehyde (4%) in 0.1 M phosphate buffer. Tissue samples were washed three times in PBS. Sections were dehydrated through a graded ethanol series (70%, 80%, 90%, 95%, 5 min each) and then twice in anhydrous ethanol (5 min each). Sections were cut at 30 μm thickness. The anatomical location of the SNpc was defined according to the Paxinos atlas. Tissue sections or treated SH-SY5Y cells were fixed in 4% paraformaldehyde, permeabilized with 0.25% Triton X-100 in PBS, and blocked for 2 h in PBST containing 1% BSA, 10% normal goat serum, and 0.3% glycine. Samples were incubated overnight with anti-TH (1:200, Abcam, USA), anti-Grb2 (1:200, Abcam, USA), anti-*p*-ERK (1:200, Proteintech, China), anti-TNFR1 (1:200, Proteintech, China), anti-p-P65 (1:200, Abcam, USA) primary antibodies, followed by appropriate secondary antibodies goat anti-rabbit IgG (H + L) (1:500, Invitrogen, Alexa Fluor 488). Finally, slides were coverslipped with DAPI mounting medium and imaged using fluorescence microscopy.

#### Immunohistochemistry (IHC)

The brain tissue was sectioned at a thickness of 5 μm, and DAB was used to stain the sections. Place the slices in 1% hydrogen peroxide and then incubate at 4°C with the blocking solution and anti-TH (1:100, Abcam, USA) reagent. Then wash the slices and incubate them with goat anti-rabbit secondary antibodies (1:500, Invitrogen, Alexa Fluor 488). In addition, incubate the slices with DAB dye to dry them and seal them with DPX. SNpc-containing sections were systematically sampled (every 3rd section; 8 sections/mouse) using Paxinos coordinates for stereological quantification. Olympus VS120 slide scanning (20× objective; 3 z-stacks, 5 μm intervals) generated digital images for unbiased cell counting, executed and verified double-blind by two investigators.

#### Hematoxylin-eosin (HE) staining

Tissue samples of murine cardiac, hepatic, pulmonary, and renal organs were fixed in 4% paraformaldehyde for 48 h. Following fixation, specimens were embedded in paraffin, sectioned into 5 μm-thick slices, and subjected to HE staining for routine histological examination.

#### Enzyme linked immunosorbent assay (ELISA)

Brain tissues and cell supernatants were collected. Concentrations of TNF-α, IL-1β, and IL-6 were measured using ELISA kits (MULTI SCIENCES BIOTECH, Wuhan, China) following the manufacturer’s instructions.

#### Quantitative reverse transcription polymerase chain reaction (RT-qPCR)

Total RNA was extracted using the FastPure Cell/Tissue Total RNA Isolation Kit V2 (Vazyme, China) and reverse-transcribed into cDNA with HiScript III RT SuperMix (+gDNA wiper) (Vazyme, China). RT-qPCR was performed on an Applied Biosystems 7500 system (Thermo Fisher Scientific, USA) using ChamQ Universal SYBR qPCR Master Mix (Vazyme, China), with primers from GENEray Biotech (Shanghai, China). β-actin served as the endogenous control, and relative gene expression was calculated via the 2−ΔΔCt method. The PCR amplification protocol consisted of four stages: (1) initial denaturation at 95°C for 30 s; (2) denaturation at 95°C for 5 s; (3) annealing/extension at 60°C for 30 s, repeated for 39 cycles; and (4) final extension at 65°C for 15 s. The primer sequences employed for PCR amplification are provided in Table S1.

#### Western blot (WB)

SN tissues or SH-SY5Y cells were lysed in RIPA buffer containing phosphatase/protease inhibitors, and protein extracts were collected. Protein concentrations were determined using a BCA assay kit (Beyotime, P0012, China). Samples were denatured at 95°C for 5 min and stored at −20°C. Proteins were separated by SDS-PAGE based on molecular weight, transferred to PVDF membranes, and blocked with 5% non-fat milk for 1 h at RT. Membranes were incubated overnight at 4°C with primary antibodies against TH (1:1000, Abcam, USA), α-Syn (1:1000, Abcam, USA), TNFR1 (1:1000, Proteintech, China), P65 (1:1000, Abcam, USA), P-P65 (1:1000, Abcam, USA), Ras (1:3000, Abcam, USA), ERK (1:2000, Proteintech, China), *p*-ERK (1:2000, Proteintech, China), TNF-α (1:1000, Proteintech, China), Bax (1:1000, Proteintech, China), Bcl-2 (1:1000, Proteintech, China), Caspase-3 (1:1000, Proteintech, China), Cleaved Caspase-3 (1:1000, Proteintech, China), and GAPDH (1:3000, Abcam, USA) (Table S2). After washing with TBST (0.1% Tween 20), membranes were incubated with secondary antibodies (1:3000, Proteintech, China) for 1 h. Protein bands were analyzed using ImageJ. Full-length blots/gels are presented in Figure S2.

#### Molecular docking

Molecular docking was performed using AutoDock Vina. Target protein structures were retrieved from the RCSB Protein DataBank (RCSB Protein DataBank：www.rcsb.org), and compound MOL2 files from PubChem. Proteins were prepared by removing water molecules, adding polar hydrogens, and merging nonpolar hydrogens. Binding affinity was evaluated from docking results visualized with PyMOL; binding energies < −5 kcal/mol indicated strong interactions.

#### Treatment with TNFR inhibitors and bioactive compounds

To verify the potential connection between TNF/NF-κB and Ras/ERK, we observed the activation changes of the two pathways and the therapeutic effect of ZGCDS after treatment with the TNFR1 inhibitor R-7050 (MCE, China, HY-110203) *in vitro*. Furthermore, to verify the role of the key active ingredients, we treated SH-SY5Y cells with active substances in the MPP^+^ cell model. The representative compounds are apigenin and bidemethoxy curcumin. Based on the dose-response curve obtained by CCK-8 determination, the optimal non-cytotoxic and effective concentrations were selected for subsequent functional studies. The experiment was divided into normal serum group (Con), MPP+ model group (Mod), AP treatment group (AP), and BDMC treatment group (BDMC).

### Quantification and statistical analysis

The details of each experiment can be found in the legends and methods. Statistical analysis of the experimental data was conducted using GraphPad Prism (version 9.5.0, California, USA). One-way analysis of variance was used for intergroup comparisons, followed by Tukey post hoc tests. Data are expressed as standard deviation (SD) ± mean. *In vitro* experiments, the value of n indicates the number of independent experimental repetitions. In *in vivo* experiments, the value of n corresponds to the number of animals used. *p* < 0.05 for the difference was statistically significant significance level: (∗*p* < 0.05, ∗∗*p* < 0.01, ∗∗∗*p* < 0.001, n.s. indicates no significance. ∗ represents comparison with the MPTP/MPP^+^ group, and # represents comparison with the Con group.

## Supplemental information

Document S1. Figures S1, S2, Tables S1, and S2

Table S3. All chemical components of ZGCD, ZGCD-CS and Con

Table S4. Screen important targets

Table S5. The result table of KEGG enrichment analysis
