# Supplementary material for: Zhigancao decoction alleviates Parkinson’s disease via inhibiting TNF/NF-κB and Ras/ERK-mediated neuroinflammation and apoptosis
Source: iScience. 2025 Dec 18;29(1):114489. doi: 10.1016/j.isci.2025.114489 (PMC12818266; doi:10.1016/j.isci.2025.114489)
Supplement: Document S1. Figures S1, S2, Tables S1, and S2 [file mmc1.pdf]

iScience, Volume *29*

Supplemental information

Zhigancao decoction alleviates Parkinson's disease via inhibiting TNF/NF-κB and Ras/ERK-mediated neuroinflammation and apoptosis

Jiakang Zhang, Xinlang Yu, Yuan Fang, Wenshan Li, Qi Cui, Xiaoyu Liu, Yanjie Jiang, Yin Zhang, Chengcheng Xu, Xin Sun, and Yan Lu

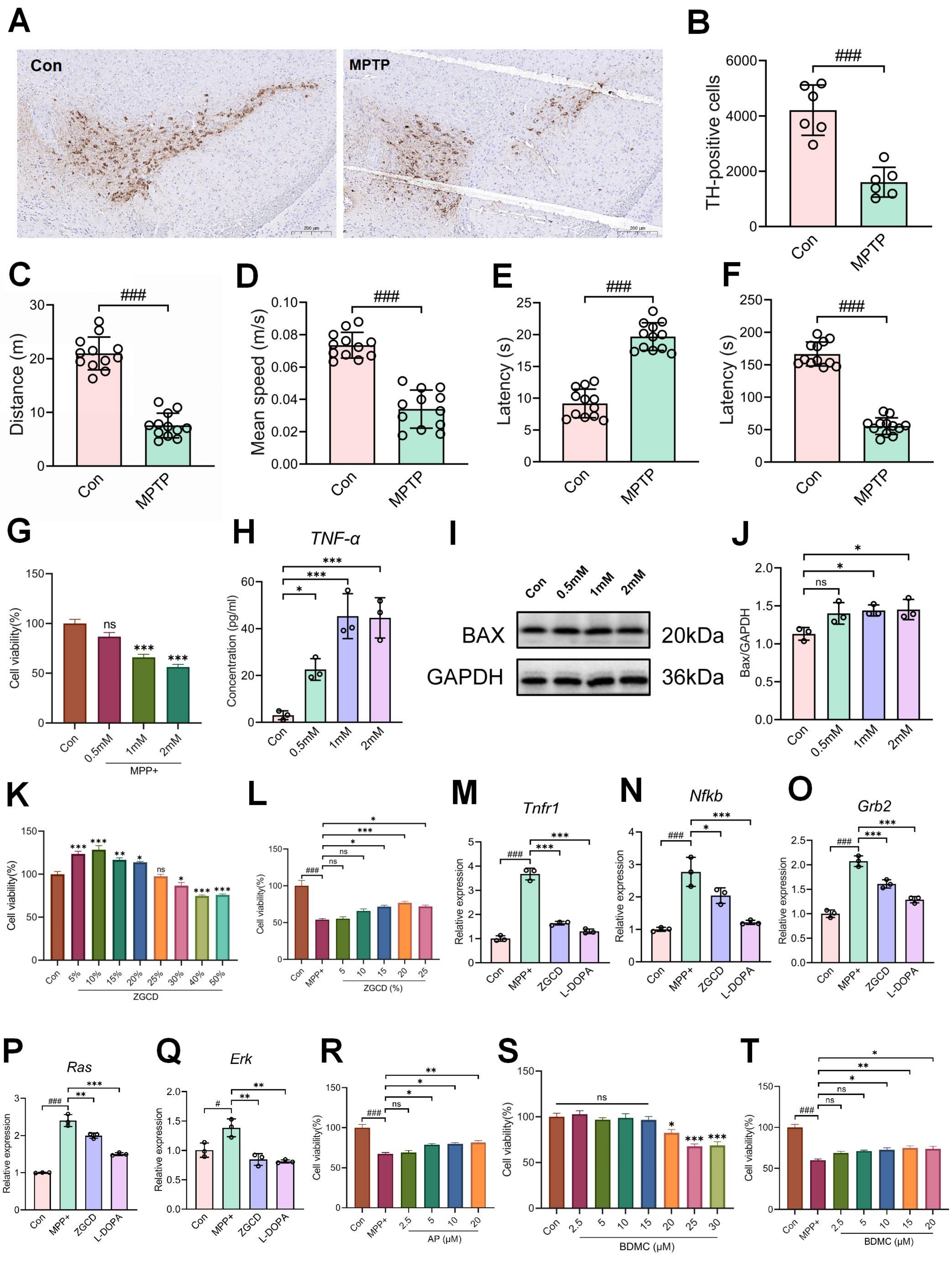

Fig. S1. MPTP successfully induced the PD model, and the concentration selection of MPP$^+$, ZGCDS, AP, and BDMC. ZGCDS significantly inhibited the expression of genes in the TNF/NF-κB and Ras/ERK signaling pathways.Validation of the MPTP mouse model, the evidence for the selection of MPP$^+$, ZGCDS, AP and BDMC and detection of pathway-related gene expression in in vitro experiments. (A) IHC analysis of TH expression in SNpc.

Scale bar, 200 μm. (B) Quantitative analysis of TH-positive neurons in SNpc (n=6). (C) The total 5 min walking distance of the mice was specified in the open field test (n=12). (D) The average speed of the mice referred to in the open field test (n=12). (E) The pole test recorded the time required for the mice to come down from the pole (n=12). (F) The time required for mice to walk on a cylindrical rotating rod was recorded in the rotarod test (n=12). (G) The cell viability of SH-SY5Y cells treated with different concentrations of MPP^+ for 24 h was determined by CCK-8. (H) ELISA was used to detect the expression level of TNF-α in SY5Y cells at different concentrations of MPP^+ (n=3). (I-J) WB analysis of Bax expression in SH-SY5Y cells at different MPP^+ concentrations (n=3). (K) CCK-8 was used to detect the cell viability of SH-SY5Y cells treated with different concentrations of ZGCDS for 48 h. (L) CCK-8 was used to detect the 24 h cell viability of SH-SY5Y cells treated with MPP^+ at different concentrations of ZGCDS. (M-Q) Representative expression of *Tnfr1* (M), *Nfkb* (N), *Grb2* (O), *Ras* (P) and *Erk* (Q) detected by RT-qPCR. (R) CCK-8 was used to detect the 24 h cell viability of SH-SY5Y cells treated with MPP^+ at different concentrations of AP. (S) CCK-8 was used to detect the cell viability of SH-SY5Y cells treated with different concentrations of BDMC for 24 h. (T) CCK-8 was used to detect the 24 h cell viability of SH-SY5Y cells treated with MPP^+ at different concentrations of BDMC.

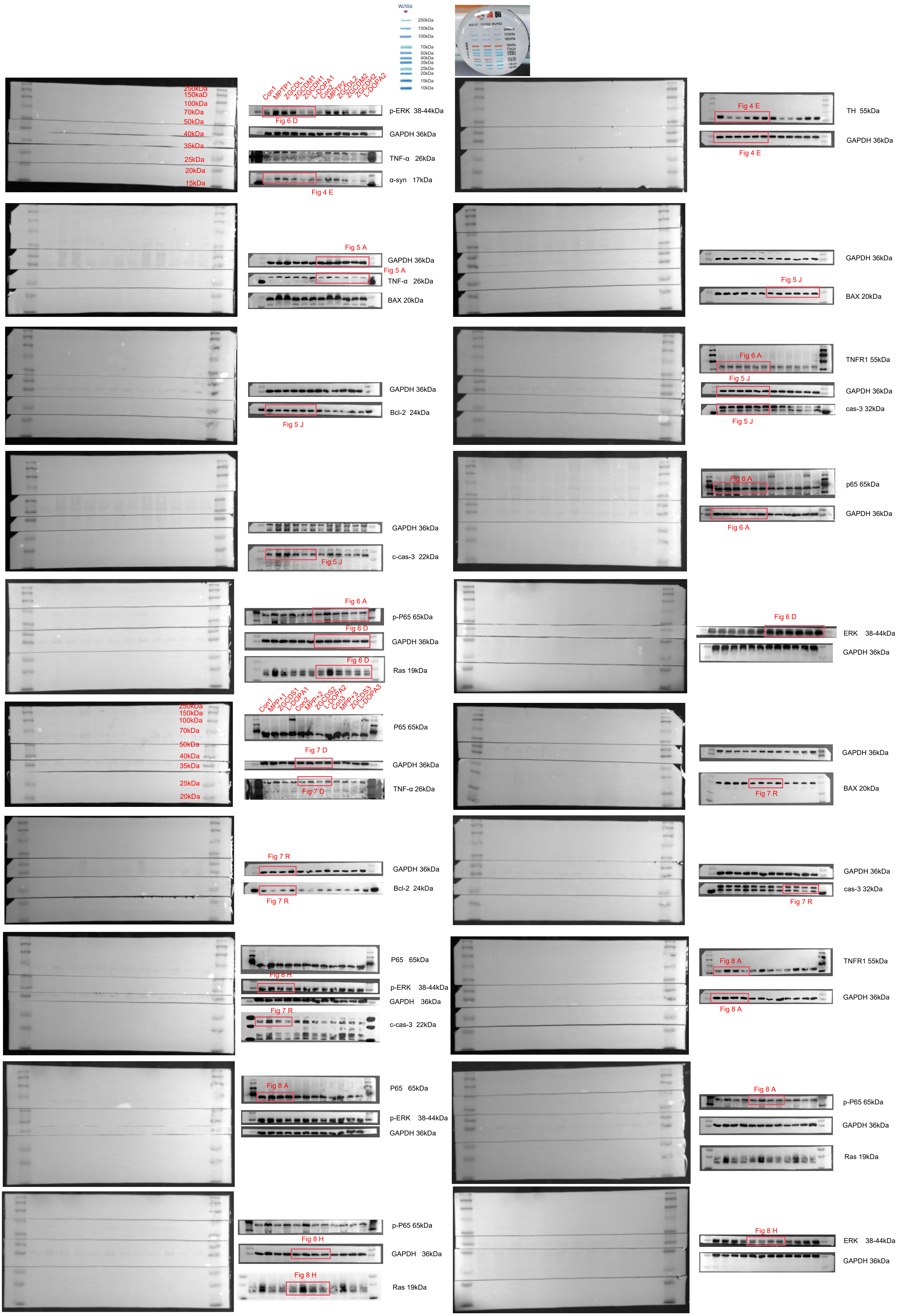
WJ106
250kDa
150kDa
100kDa
70kDa
50kDa
40kDa
35kDa
25kDa
20kDa
15kDa
10kDa
250kDa
150kaD
100kDa
70kDa
50kDa
40kDa
35kDa
25kDa
20kDa
15kDa
Con1 MPTP1 ZGCDL1 ZGCDM1 ZGCDH1 L-DOPA1 Con2 MPTP2 ZGCDL2 ZGCDM2 ZGCDH2 L-DOPA2
p-ERK 38-44kDa
Fig 6 D
GAPDH 36kDa
TNF-α 26kDa
α-syn 17kDa
Fig 4 E
Fig 4 E
TH 55kDa
GAPDH 36kDa
Fig 4 E
Fig 5 A
GAPDH 36kDa
Fig 5 A
TNF-α 26kDa
BAX 20kDa
GAPDH 36kDa
Fig 5 J
BAX 20kDa
GAPDH 36kDa
Bcl-2 24kDa
Fig 5 J
Fig 6 A
TNFR1 55kDa
Fig 5 J
GAPDH 36kDa
cas-3 32kDa
Fig 5 J
GAPDH 36kDa
c-cas-3 22kDa
Fig 5 J
Fig 6 A
p65 65kDa
GAPDH 36kDa
Fig 6 A
Fig 6 A
p-P65 65kDa
Fig 6 D
GAPDH 36kDa
Fig 6 D
Ras 19kDa
Fig 6 D
ERK 38-44kDa
GAPDH 36kDa
250kDa
150kDa
100kDa
70kDa
50kDa
40kDa
35kDa
25kDa
20kDa
Con1 MPP+1 ZGCDS1 L-DOPA1 Con2 MPP+2 ZGCDS2 L-DOPA2 Con3 MPP+3 ZGCDS3 L-DOPA3
P65 65kDa
Fig 7 D
GAPDH 36kDa
TNF-α 26kDa
Fig 7 D
GAPDH 36kDa
BAX 20kDa
Fig 7 R
Fig 7 R
GAPDH 36kDa
Bcl-2 24kDa
Fig 7 R
GAPDH 36kDa
cas-3 32kDa
Fig 7 R
P65 65kDa
Fig 8 H
p-ERK 38-44kDa
GAPDH 36kDa
Fig 7 R
c-cas-3 22kDa
Fig 8 A
TNFR1 55kDa
GAPDH 36kDa
Fig 8 A
Fig 8 A
P65 65kDa
p-ERK 38-44kDa
GAPDH 36kDa
Fig 8 A
p-P65 65kDa
GAPDH 36kDa
Ras 19kDa
p-P65 65kDa
Fig 8 H
GAPDH 36kDa
Fig 8 H
Ras 19kDa
Fig 8 H
ERK 38-44kDa
GAPDH 36kDa

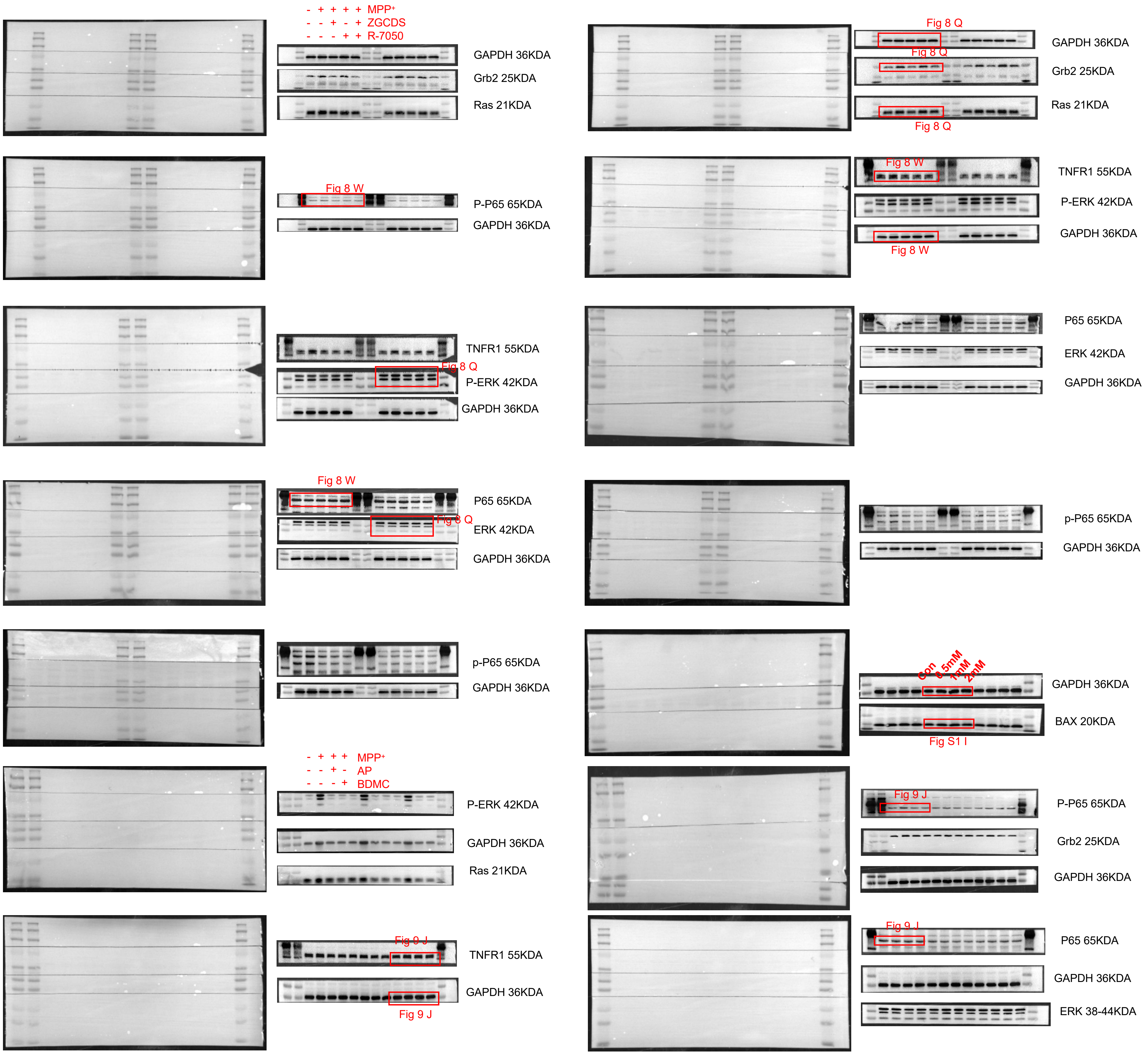

Fig. S2. Original strip diagrams of WB

Table. S1. Sequence information of RT-qPCR analysis primers.

mRNA	Species	Primer sequence 5'-3'
Tnfr1	Mouse	F：GCTGTTGCCCCTGGTTATCT
		R：ATGGAGTAGACTTCGGGCCT
	Homo	F：CGAGGATGAGGGACGCTATG
		R：GCACAGGAGTGCCAAGTTTC
Nfkb	Mouse	F：GGAGGCATGTTCGGTAGTGG
		R：CCCTGCGTTGGATTTCGTG
	Homo	F：AATGGGCTACACCGAAGCAA
		R：TTGCGGAAGGATGTCTCCAC
Grb2	Mouse	F：GCAAAATCCCCAGAGCCAAG
		R：TCCAAACTTGACGGACAGGG
	Homo	F：CCGTGAACCGGAACGTCTAA
		R：TTAAATCCAACGCCCCCTCC
Ras	Mouse	F：GCGCCTTGACGATACAGCTAA
		R：TACACAAAGAAAGCCCTCCCC
	Homo	F：GTGGTGGTCCTGCTGACAAA
		R：ACCACCACCCCAAAATCTCAA
Erk	Mouse	F：CCAAACAAGCGCATCACAGT
		R：CAGCTCCATGTCGAAGGTGA
	Homo	F：AGTACATCCACTCCGCCAAC
		R：AGCCACATACTCCGTCAGGA
TNF-α	Mouse	F：ATCCGCGACGTGGAACTG
		R：ACCGCCTGGAGTTCTGGAA
	Homo	F：CCTCTCTCTAATCAGCCCTCTG
		R：GAGGACCTGGGAGTAGATGAG
IL-1β	Homo	F：ATGATGGCTTATTACAGTGGCAA
		R：GTCGGAGATTCGTAGCTGGA
IL-6	Homo	F：ACTCACCTCTTCAGAACGAATTG
		R：CCATCTTTGGAAGGTTCAGGTTG
Bax	Mouse	F：GGAGACACCTGAGCTGACCTTG
		R：GCTCCATATTGCTGTCCAGTTCATC
	Homo	F：TTCTGACGGCAACTTCAACT
		R：CAGCCCATGATGGTTCTGAT
Bcl-2	Mouse	F：GGACTTGAAGTGCCATTGGT
		R：AGCCCCTCTGTGACAGCTTA
	Homo	F：GGATAACGGAGGCTGGGATG
		R：TGACTTCACTTGTGGCCCAG
Caspase-3	Mouse	F：GAGCTTGGAACGGTACGCTA
		R：GAGTCCACTGACTTGCTCCC
	Homo	F：AGATGGTTTGAGCCTGAGCA
		R：GTGCGTATGGAGAAATGGGC
β-actin	Mouse	F：GATCAGCAAGCAGGAGTACGA
		R：GGGTGTAAAACGCAGCTCA

	Homo	F：CTCGCCTTTGCCGATCC<br>R：ATCCTTCTGACCCATGCCC

Table. S2. Primary and secondary antibodies.

Antibodies	Sources	Identifier	RRID
Rabbit monoclonal to TH	Abcam	ab137869	AB_2801410
Rabbit monoclonal to α-Syn	Abcam	ab138501	AB_2537217
Rabbit monoclonal to Ras	Abcam	ab52939	AB_2121042
Rabbit monoclonal to Grb2	Abcam	ab32111	AB_2113026
Rabbit polyclonal to ERK	Proteintech	11257-1-AP	AB_2139822
Rabbit polyclonal to p-ERK	Proteintech	28733-1-AP	AB_2881202
Rabbit polyclonal to TNF-α	Proteintech	17590-1-AP	AB_2271853
Rabbit polyclonal to TNFR1	Proteintech	21574-1-AP	AB_10734433
Rabbit monoclonal to P65	Abcam	ab32536	AB_776751
Rabbit monoclonal to p-P65	Abcam	ab76302	AB_1524028
Rabbit polyclonal to Bax	Proteintech	50599-2-Ig	AB_2061561
Rabbit polyclonal to Bcl-2	Proteintech	26593-1-AP	AB_2818996
Rabbit polyclonal to Caspase-3	Proteintech	19677-1-AP	AB_10733244
Rabbit polyclonal to Cleaved-Caspase-3	Proteintech	25128-1-AP	AB_3073913
Rabbit monoclonal to GAPDH	Abcam	ab181602	AB_2630358
Anti-rabbit IgG, HRP-linked Antibody	Proteintech	SA00001-2	AB_2722564
Anti-rabbit IgG, AF488 antibodies	Invitrogen	A-11094	AB_221544
